# Tracking *Batrachochytrium dendrobatidis* Infection Across the Globe

**DOI:** 10.1007/s10393-020-01504-w

**Published:** 2020-11-17

**Authors:** Federico Castro Monzon, Mark-Oliver Rödel, Jonathan M. Jeschke

**Affiliations:** 1 Institute of Biology, Freie Universität Berlin, Königin-Luise-Str. 1-3, 14195 Berlin, Germany; 2 Leibniz-Institute of Freshwater Ecology and Inland Fisheries, Müggelseedamm 310, 12587 Berlin, Germany; 3 Berlin-Brandenburg Institute of Advanced Biodiversity Research, Königin-Luise-Str. 2-4, 14195 Berlin, Germany; 4 Museum für Naturkunde – Leibniz Institute for Evolution and Biodiversity Science, Invalidenstr. 43, 10115 Berlin, Germany

**Keywords:** Emerging infectious disease, *Batrachochytrium dendrobatidis*, Amphibian pathogen, Chytrid, Chytridiomycosis, Systematic review

## Abstract

Infection records of *Batrachochytrium dendrobatidis* (*Bd*), a pathogen that has devastated amphibian populations worldwide, have rapidly increased since the pathogen’s discovery. Dealing with so many records makes it difficult to (a) know where, when and in which species infections have been detected, (b) understand how widespread and pervasive *Bd* is and (c) prioritize study and management areas. We conducted a systematic review of papers and compiled a database with *Bd* infection records. Our dataset covers 71 amphibian families and 119 countries. The data revealed how widespread and adaptable *Bd* is, being able to infect over 50% of all tested amphibian species, with over 1000 confirmed host species and being present in 86 countries. The distribution of infected species is uneven among and within countries. Areas where the distributions of many infected species overlap are readily visible; these are regions where *Bd* likely develops well. Conversely, areas where the distributions of species that tested negative overlap, such as the Atlantic Coast in the USA, suggest the presence of *Bd* refuges. Finally, we report how the number of tested and infected species has changed through time, and provide a list of oldest detection records per country.

**Electronic supplementary material:**

The online version of this article (10.1007/s10393-020-01504-w) contains supplementary material, which is available to authorized users.

## Introduction

The parasitic chytrid fungus *Batrachochytrium dendrobatidis* (*Bd*) has been associated with amphibian declines across the world. *Bd* stands out from other emerging diseases by having driven several of its host species to extinction and gaining a notorious place as the disease with the greatest impact on vertebrate biodiversity (Skerratt et al. 2007; Scheele et al. 2019). The dramatic impact *Bd* has on amphibian communities has stimulated intense research and has driven a quest to understand which amphibian communities or species are susceptible to infection, where has infection been detected and since when is infection present in an area. Answering these questions “who, where and when” presents a great challenge.

In the past, efforts have been undertaken to compile and analyze published data on infection (Fisher et al. 2009; Olson et al. 2013; Olson and Ronnenberg 2014) and to maintain a database that kept track of new reports (www.bd-maps.net). Olson and Ronnenberg (2014) have shown *Bd* infections in 71 countries and 695 species, a number often cited to illustrate the scale of the pandemic. This compilation of data has produced valuable insight into how widespread and pervasive *Bd* is, revealing the distribution of infected species and showing how traits and environmental conditions are associated with infection. Unfortunately, the *Bd*-maps database is no longer functional.

Over time, new reports of infection have appeared from areas where this pathogen had not previously been detected (e.g., Conradie et al. 2016; Seimon et al. 2015; Bletz et al. 2015). These reports appear as we fill gaps in our knowledge, but also as *Bd* distribution changes over time. As environmental conditions change so does the severity of *Bd* infection, the species that it infects and the range that is suitable for infection to develop (Burrowes et al. 2004; Rohr and Raffel 2010; Bosch et al. 2018). Evolutionary and demographic processes such as migration, hybridization and adaptation can also bring previously unexposed hosts into contact with *Bd* or new and more virulent *Bd* lineages (Rosenblum et al. 2013; Savage and Zamudio 2016). With the involuntary aid of humans, *Bd* is known to move between continents and over areas where *Bd* cannot develop (Mazzoni et al. 2003; Weldon et al. 2004). *Bd* lineages, which are seemingly innocuous to amphibians within their known range, can be lethal for amphibians from regions that have not been in contact with that lineage and are evolutionarily naïve to it (Becker et al. 2017). The arrival of a *Bd* lineage to a new location where a different *Bd* lineage was already present brings about the possibility of hybridization. Two such hybrids have been found (Byrne et al. 2019), and there are observations that hybrids can be more virulent than the parental species (Greenspan et al. 2018). This last point is highly relevant in light of the recent discovery of *Bd* lineages in regions where they were not known to be present (Byrne et al. 2019).

As infection changes and new data appear, it becomes difficult to keep track of which species have been found with infection and when and where these species were sampled. New data become available but are not currently compiled in a database that allows producing and updating summary statistics. The number of infected species, for example, has not been updated since Olson and Ronnenberg’s (2014) work. Other summary statistics, such as the number and percentage of infected species across all countries and amphibian families, can only be produced with data from several studies. In this study, we assembled a database where we compiled records of infection. This allowed us to produce summary statistics that reflect how widespread and adaptable *Bd* is. We particularly seek to understand in which countries, regions and species *Bd* has been detected, how infection distribution changed over time, and if there are clear patterns in the distribution of infected species.

## Methods

We conducted a systematic review of available literature to study spatial and temporal patterns of *Bd* infection in wild amphibian populations. We followed the PRISMA guidelines for systematic review of data (Moher et al. 2010) in order to improve study repeatability: future studies can follow the same procedure to obtain relevant sources and thus extend the dataset based on the same method. We are painfully aware that, despite the best intentions of researchers, online databases often become non-functional. We, therefore, offer our data in this paper (see Supplementary Table 1), as a comma-separated values file (csv), a cross-platform standard that has been in use for almost 50 years and is likely to remain stable in years to come.

We restricted our sources to peer-reviewed publications indexed in the Web of Science (http://isiknowledge.com), with the exception of one non-indexed journal, Herpetological Review, because in one section of this journal many *Bd*-related reports are published (see data by Olson et al. 2013). Thus, our study does not include data from gray literature, unpublished accounts of infection, conference contributions and publications that are not indexed in the Web of Science (other than Herpetological Review). We intentionally excluded data from reviews, as we focus on original primary sources. Hence, no data were extracted from any review.

We searched the Web of Science on May 4, 2020, using the following string: (chytrid* OR batrachochytrium OR fungal) AND (amphibian* OR frog* OR salamander* OR anuran* OR urodelan* OR caecilian*).

We excluded news items, reviews, editorial material, letters, corrections, meeting abstracts and book chapters. Additionally, we looked for all Herpetological Review *Bd*-articles up to the first volume of this journal in 2020. We searched articles containing the words “batrachochytrium” or “chytrid.” We selected relevant papers from the Web of Science and Herpetological Review according to the following criteria: (1) amphibian hosts were tested for *Bd*; (2) *Bd* presence or absence was ascertained by means of histological or molecular methods, or by isolating the pathogen; (3) the conditions were not intentionally manipulated to alter infection and (4) the amphibian hosts were collected in the field.

As we focus on *Bd* here, data from *Bsal* and other pathogens are not included in our dataset. Further, we did not include papers that reported the presence of *Bd* on the basis of environmental DNA in non-amphibian hosts. Similarly, we excluded papers that diagnosed *Bd* solely on the presence of lesions on amphibians. There are conflicting reports on the accuracy of visual inspection to diagnose *Bd* infection. Knapp and Morgan (2006) report an 86% accuracy, whereas Navarro-Lozano et al. (2018) report only 40%, and Padgett-Flohr and Goble (2007) report 36% accuracy.

We excluded papers that used amphibians raised in captivity, specimens that came from the amphibian trade or that were tested for infection after being in an experimental setup. If only part of the data met the selection criteria, only these data were included in the database. Studies that tested specimens preserved in ethanol or formalin were included if they fitted the inclusion criteria.

Based on these criteria, we retained 554 publications for data extraction. For every record, we tried to assess the species names, the country in which the species were collected, first and last year in which the study was conducted, first and last date in which each species was sampled, first and last date in which each species was found with infection, whether or not the samples came from a preserved collection and whether the test turned out to be positive for *Bd*. A test was taken as positive if respective authors identified *Bd* as the causal agent of infection by means of histological, molecular analysis or if the authors isolated and cultured the pathogen.

The species names, as reported, were matched with those of the Amphibian Species of the World database (Frost 2020), with the last update on May 6, 2020. Hybrids, kleptons, amphibians with unresolved taxonomic status and amphibians that could not be matched with a species as per Frost (2020) were recorded but not counted as species in the data analysis. We calculated the total number of species sampled and infected, and estimated the percentage of species that have been found with infection. Data on species conservation status and distribution were extracted from the IUCN Red List database (https://www.iucnredlist.org); these data were last checked on May 6, 2020.

## Results

Our literature search produced 554 relevant papers according to our selection criteria. We found data from all continents where amphibians exist, encompassing 119 countries. In 61 of these countries, 10 or more amphibian species were sampled and tested for *Bd,* a number that we consider adequate for analysis. Few species from countries like Laos and Tanzania are present in the data, making it difficult to draw conclusions on how widespread and prevalent *Bd* is in those countries (see Supplementary Table 2 for details).

Variation in the number of sampled species among countries was expected given the latitudinal patterns of diversity. Not surprisingly, more species were sampled in countries with greater amphibian diversity. However, a non-proportionally large number of species came from North America (particularly the USA), whereas relatively few species came from the equatorial regions of South America and from Madagascar (Supplementary Table 2 for details). Similarly, large areas in continental Africa, India, Borneo and Papua New Guinea remain understudied both in terms of the number of untested species in those areas and in terms of the percentage of untested species (Supplementary Figure 1 for details). In Africa and Southeast Asia, few studies testing for *Bd* have been conducted and several years have passed since the last study in some of these areas (Supplementary Figure 2).

**Figure 1:**
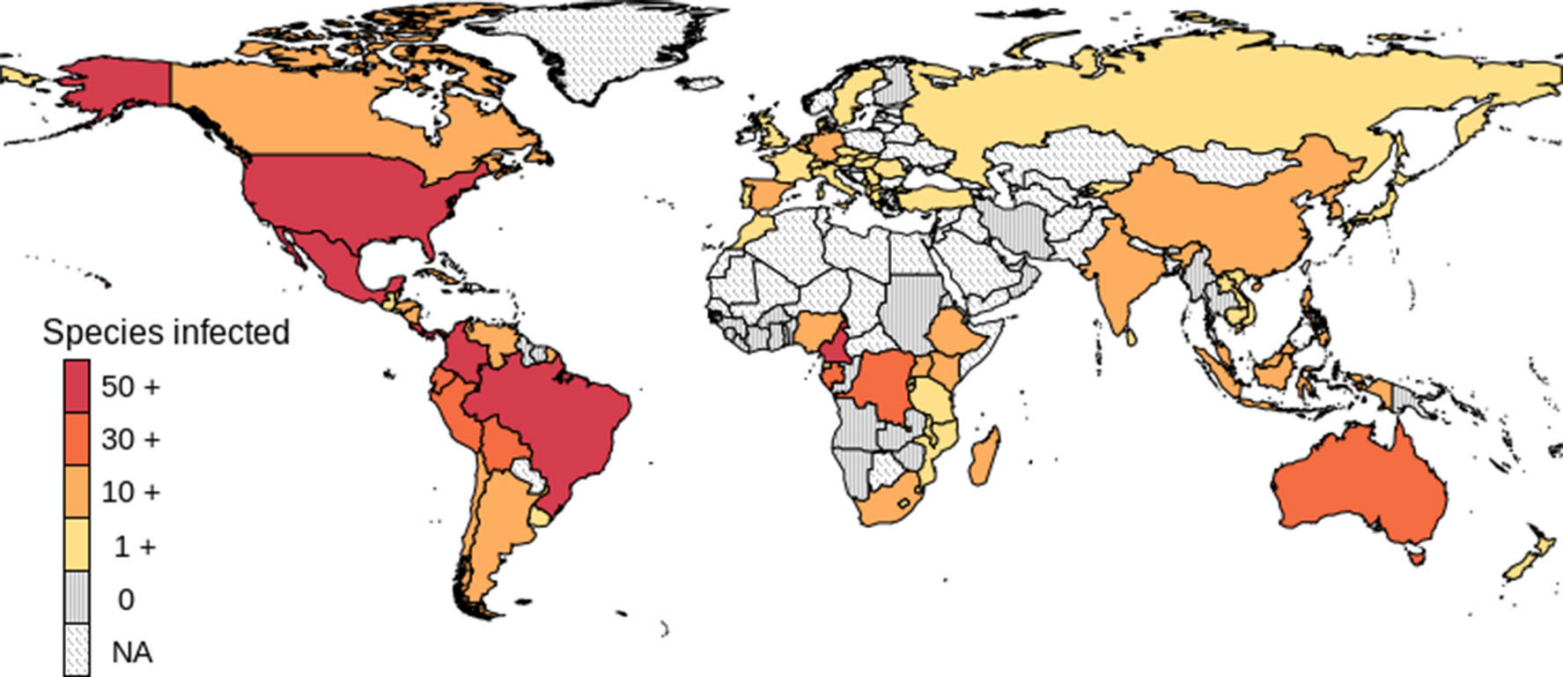
Spatial distribution of the number of *Bd*-infected amphibian species in each country. For specific data points see Supplementary Table 2.

*Bd*-infection was found in 86 of the 119 sampled countries (Fig. 1). Infection was also present in most countries where at least 10 species were sampled and in the 10 countries with the largest number of amphibian species in the world (Supplementary Table 2). Similarly, infection has been found in the 10 countries with the largest number of threatened amphibian species (IUCN status = critically endangered, endangered or vulnerable). The data included amphibians from 71 families. Infection was reported for 62 (87%) of these families. Within amphibian families, the number of sampled species varied, an expected result given the differences in family size. However, some moderately large amphibian families such as Ceratobatrachidae or Brachycephalidae were not well represented in the data. In 34 families, at least 10 species were sampled, and infection was reported in all these well-sampled families (Supplementary Table 3).

### Number of Infected Species and Prevalence of Infection Globally

We used two summary statistics for evaluating the prevalence of infection: the number of infected species in a country and the percentage of sampled species that tested positive per country. In papers included in this study, a total of 1966 species were tested for *Bd* of which 1062 (54%) were found with infection.

### Tracking the Infection Over Time

**Figure 2:**
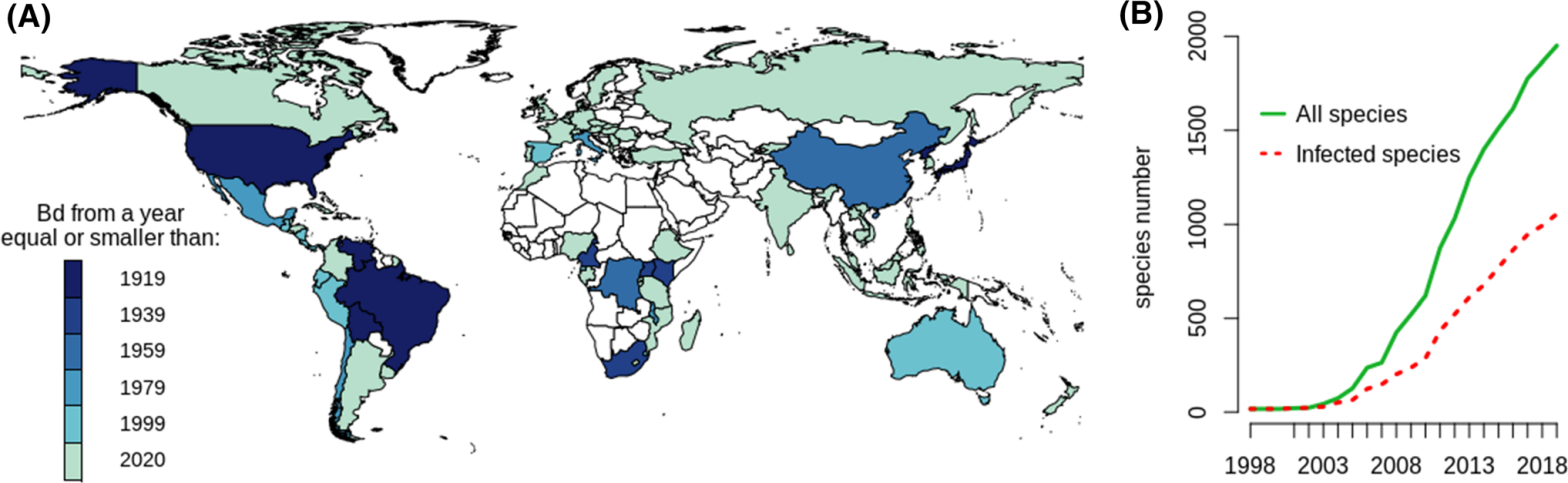
Temporal patterns in Bd infections. **A** Earliest infection records. **B** Cumulative count of sampled and infected amphibian species over the world reported in papers published since 1998. All data before 1999 comes from preserved specimens. For specific data points see Supplementary Tables 1 & 4.

We compiled a list of first detection records for all countries in which infection has been detected (Supplementary Table 4) including records of infection on dates prior to 1999 from 24 countries. The compiled list shows *Bd* records from North America, South America, Asia and Africa from more than 80 years ago. Records from preserved specimens show that by 1980 *Bd* was also present in Central America and Europe (Fig. 2A). The total number of tested species, infected and otherwise, has grown over time as new species are being tested (see Fig. 2B). Within tested species, the percentage of species that have been reported with infection was about half (54%) which is only slightly higher than the percentages reported by Olson et al. (2013) and Fisher et al. (2009): they reported 42% and 50%, respectively.

### Geographical Range of Infected Species

**Figure 3:**
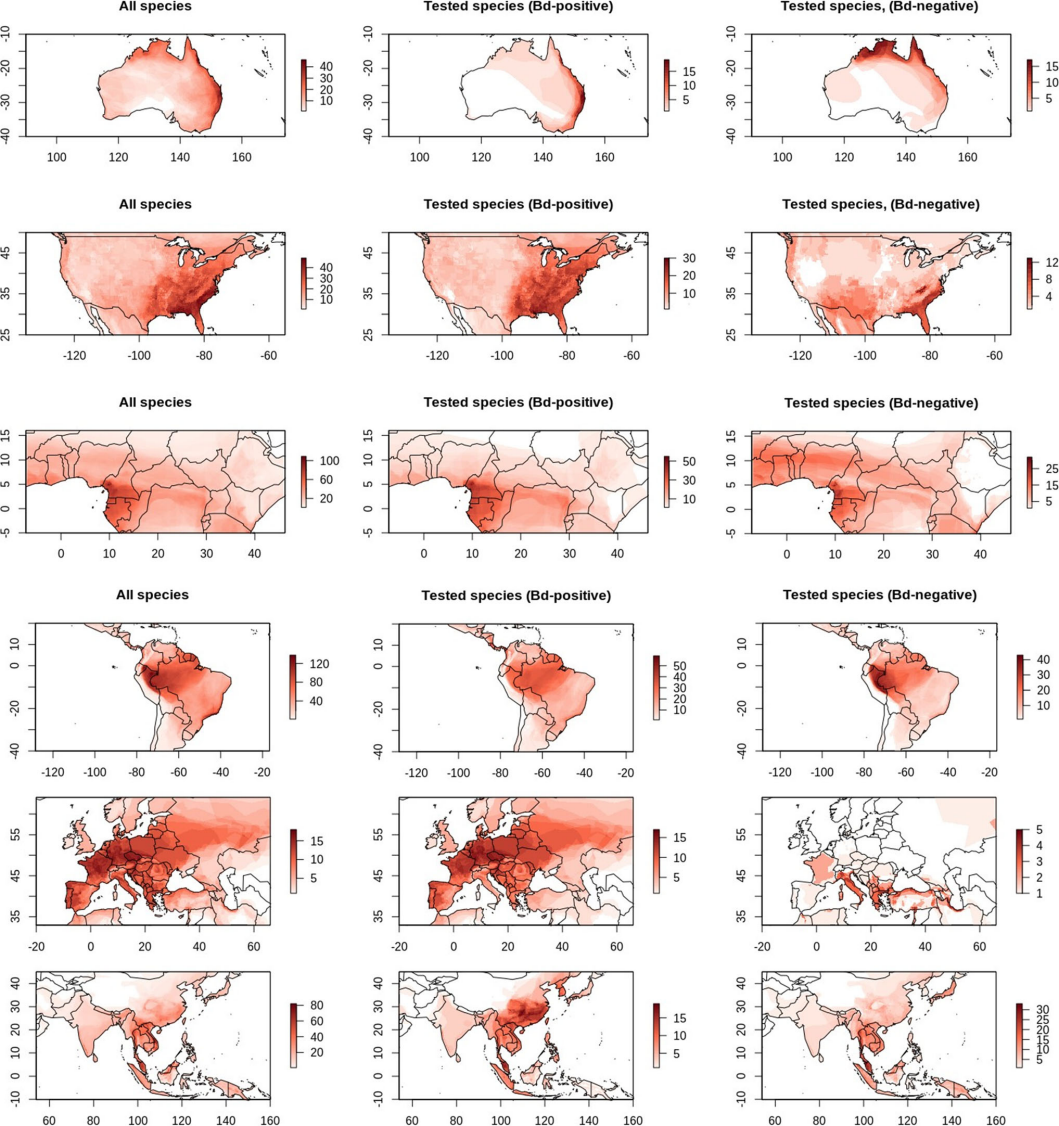
Amphibian species distribution in six regions of the world. The leftmost column shows all species, the middle column only species that tested positive for *Bd* and the rightmost column only species that have only tested negative. Color intensity correspond to the number of species in a region, scales vary between plots. Notice patterns in the distribution of species that have been found with and without infection (Color figure online).

Regions where the spatial distribution of *Bd*-infected species is different from the overall species distribution reveal interesting patterns (Fig. 3). In Australia, for example, species in which infection has been detected concentrate along the east coast in regions farther from the equator. In the USA, the distribution of species in which infection has been detected, roughly mirrored species richness patterns. However, species that have not tested positive concentrate over an area that extends over the Atlantic Coast in the USA from Mississippi to Virginia (Fig. 3). The range of infected species that went across national borders also revealed areas or countries where infection might not have been detected or directly tested for, but where a large number of susceptible species reside. One such area can be seen along the lowlands in Cameroon, producing a corridor that stretches eastward all the way to Uganda (Fig. 3). Differences in the distribution of infected and non-infected species can also be seen in other areas of the world. These include a concentration of host species over China and the Korean Peninsula, and a concentration of resistant species in southern Europe along the Italian peninsula, Greece and Turkey (Fig. 3).

### Associations Between *Bd* Susceptibility of Amphibian Taxa and Geographical Distribution

We found marked differences in the percentage of species with infection among amphibian families. These differences are often associated with a high species infection prevalence in an area as many amphibian families have restricted ranges due to biogeographical processes. On one end of the spectrum, high species infection rates were reported in families from South America such as Telmatobiidae (95%) and Hylodidae (85%). On the other end, low species infection rates were reported in families from Southeast Asia such as Megophryidae (7%) and Hynobiidae (17%); the latter only being sampled in South East Asia. Variation among larger families did not reach such extreme values and was closer to 50% (Supplementary Table 3). Examples of this include Plethodontidae (47%), Bufonidae (50%) Craugastoridae (50%), the Hylidae (70%) and Microhylidae (30%).

## Discussion

Our results illustrate the spread of *Bd* over time and provide an important update of previous work addressing where, when and in which species *Bd* infection has been found (Fisher et al. 2009; Olson et al. 2013). While Olson and Ronnenberg (2014) reported 695 species that have been infected with *Bd*, we found 1062 infected species. This does not necessarily indicate an increase in *Bd* prevalence or an increase in its distribution; the accumulation of data and the growth in the research field could also produce such an increase. Indeed, the percentage of species that have been found with infection does not show a clear temporal trend. However, the fact that the rate at which infected species accumulate does not appear to slow down suggests that we are still discovering *Bd* in previously untested locations and that we are yet to fully test most of the susceptible species and to fully understand the spatial distribution of *Bd*.

Our study shows records of infection in 86 out of 119 countries. Most of these records are relatively recent and date after 1999. It is hard to ascertain whether infection spread recently to an area or it was long present at undetectable levels; in peninsular Malaysia, for example, extensive search was conducted for years before the infection was detected (LeBlanc et al. 2014). However, early records from surrounding areas are revealing (Fig. 2). Records before 1999 were found for 24 countries, including data showing that *Bd* had spread around the world from more than a century ago. Positives have been found from 1888 in the USA, 1894 in Brazil, from 1910 in what today is North Korea and from the 1930s in Cameroon, South Africa, Kenya and Uganda (Supplementary Table 4). The presence of *Bd* positives in museum specimens across the world together with patterns of genetic diversity found previously (O’Hanlon et al. 2018) suggests there were multiple introduction events in the past. We know that *Bd* has spread with the unintentional aid of humans (Walker et al. 2008) and it probably did so in the past as well. This is particularly likely when considering the large distances that *Bd* crossed to spread across continents.

We highlight the extent of *Bd* spread up to this day and the variety of hosts that the pathogen is able to infect. This was reflected in the number and proportion of countries where infection has been detected (86/119 = 72%), the number and proportion of amphibian families with infection (62/71 = 87%) and the number and proportion of species with infection (1062/1966 = 54%). At a global scale, the number and proportion of infected species as well as its great spread showed how pervasive and adaptable *Bd* is and, indirectly, how easy it is for it to spread via one of its many amphibian hosts. When studying individual countries, we observed a high variability in the percentage of infected species. Part of this variability could be adjudicated to different sample sizes but also to latitudinal, altitudinal and environmental conditions that favor or limit the development of infection. Variation in the number of species from a family or country also reveals overlooked and unstudied locations or amphibian groups (Supplementary Table 3). The Microhylidae, for example, are relatively understudied given how speciose this family is.

When dealing with a lack of data, the distribution of species that are susceptible to *Bd* or that have not been found with infection can be useful. The distribution area of *Bd*-infected species is, in some areas, quite different from the overall distribution of species and reveals spatial patterns of infection (Fig. 3). These patterns probably correspond to geographical and environmental conditions that affect infection. The pattern in Australia, for example, roughly follows the *Bd* suitability models by Murray et al. (2011), probably reflecting the influence of environmental conditions on infection. In Asia, a large number of susceptible species concentrate in China and the Korean peninsula, which is in line with models on the suitability of *Bd* by Rahman et al. (2020). However, disagreeing with that work, we do not observe a high concentration of susceptible species in the Indian Western Ghats. *Bd* has been postulated to have its center of diversity around the Korean peninsula (O’Hanlon et al. 2018), and the pathogen is presumably well adapted to the conditions and hosts existing in the area where it evolved.

Besides areas where susceptible species concentrate, the distribution of species that have not tested positive is also revealing. In the USA, for example, a pattern is formed by the distribution of non-infected species over the Atlantic Coast of the USA from Mississippi to Virginia. This suggests again that the altitudinal profile and the environmental conditions prevalent in the region affect *Bd* infection. Another pattern, found in Central Africa, shows a high proportion of species that have been found without infection that are present in dry regions. Further, we observed a corridor-like area formed by the distribution of infected species. This area stretches from the Cameroonian lowlands all the way to the Democratic Republic of Congo. However, only small discontinuous regions within the corridor have been predicted to be suitable for *Bd* by models (Penner et al. 2013; Zimkus et al. 2020). If indeed *Bd* infection is absent or of low severity in that location, the corridor would provide a refuge for a large number of susceptible amphibian species. Equally interesting is the low concentration of susceptible species over the Amazonian region of Peru. Against our expectations, we did not observe a high number of infected species over the Atlantic forest as predicted by Becker et al. (2017).

The observed patterns should be interpreted with caution. For example, the low amphibian diversity in Europe gives great weight to individual species, and the presence or absence of infection in a single species could radically change observed patterns. We observed that species in which infection has not been detected concentrate in southern areas of Europe, mostly the Italian peninsula, Greece, the Balkans and northern Turkey. However, with the exception of the Italian peninsula, these regions are not well studied (Supplementary Figure 2), and the observed patterns might change as *Bd* is studied more intensively there as well.

*Bd* is widespread, although infection and prevalence are not necessarily high in all regions where it is found. Also, there are isolated regions like Papua New Guinea where this pathogen has not been detected (Bower et al. 2019). Constant monitoring and the establishment of proper biosecurity mechanisms can help to ensure that *Bd* does not expand its range and to react rapidly to its detection. These measures are equally important in regions where *Bd* is present in order to prevent and react to new and more virulent *Bd* strains or related pathogens such as *Batrachochytrium salamandrivorans*, as well as to react to changes in infection dynamics. Monitoring programs and strict biosecurity measures are thus highly important. *Bd* has shown how a pathogen can maintain infection for decades across the globe, and have massive detrimental effects on a wide range of hosts. The appearance of an analogous pathogen that could infect another taxonomic group, domestic animals or even humans presents a worrying scenario.

## Conclusions

*Bd* is widespread and pervasive, being able to infect more than 50% of the studied species and having more than 1000 known amphibian hosts. As of this publication, amphibians in most countries and families have been tested. However, several of them have been barely and infrequently studied. We stress the need to prioritize efforts to study and evaluate the effect that *Bd* has on amphibian populations across the world. Our data highlight amphibian-rich regions and families where *Bd* has been detected, but where only few or no species have been tested. Countries such as Laos and Tanzania cannot be properly evaluated right now (see Table 2). Similarly, there are moderately large amphibian families, such as Ceratobatrachidae or Brachycephalidae, that have not been well studied (see Table 3). We investigated the distribution of *Bd* over time, pointing out some temporal, spatial and taxonomic patterns of infection. We acknowledge the speed with which data accumulate and the need to maintain a commitment to update our dataset in the future. We extend a cordial invitation to researchers interested in using, updating or extending this dataset to join our effort to maintain data on *Bd* records.

## Electronic supplementary material

Below is the link to the electronic supplementary material.

Supplementary material 1 (DOCX 8 kb)

Supplementary material 2 (PDF 297 kb)

Supplementary material 3 (PDF 186 kb)

Supplementary material 4 (CSV 1030 kb)

Supplementary material 5 (CSV 11 kb)

Supplementary material 6 (CSV 4 kb)

Supplementary material 7 (CSV 40 kb)

Supplementary material 8 (CSV 4 kb)

## Data Availability

All data generated or analyzed during this study are included in this published article and its supplementary information files.
