# Supplementary material for: Tracking Batrachochytrium dendrobatidis Infection Across the Globe
Source: Ecohealth. 2020 Nov 17;17(3):270–9. doi: 10.1007/s10393-020-01504-w (PMC7719156; doi:10.1007/s10393-020-01504-w)
Supplement: Supplementary file 1 — Supplementary material 1 (DOCX 8 kb) [file 10393_2020_1504_MOESM1_ESM.docx]

**List of documents in supplementary materials**

| **Document** | **File name** |
| --- | --- |
| Supplementary Materials Figures 1 & 2 | 10393_2020_1504_MOESM2_ESM.pdf |
| Supplementary Materials Bibliography | 10393_2020_1504_MOESM3_ESM.pdf |
| Supplementary Materials Table 1 | 10393_2020_1504_MOESM4_ESM.csv |
| Supplement Materials Table 2 | 10393_2020_1504_MOESM5_ESM.csv |
| Supplement Materials Table 3 | 10393_2020_1504_MOESM6_ESM.csv |
| Supplement Materials Table 4 | 10393_2020_1504_MOESM8_ESM.csv |
| Supplement Materials Table 5 | 10393_2020_1504_MOESM7_ESM.csv |

**Supplementary Materials Bibliography**

A list of sources from where bibliography was extracted.

This document contains all the sources used by the tables.

**Supplementary Materials Table 1**

A comma separated value table containing all data extracted from papers.

The symbol “-” is used for missing data to avoid conflict with the iso2 code of Namibia (NA).

| **Field name** | **Description** |
| --- | --- |
| reference | Bibliography referencing the document “Supplement Materials Bibliography”. |
| wos_id | Web of Science accession number. |
| pubyear | Publication year. |
| r_genera | Genera of the species “as reported” in the source paper. |
| r_species | Species name “as reported” in the source paper. |
| order | Order of the species as per Amphibian Species of the World database. |
| superfamily | Superfamily of the species as per Amphibian Species of the World database. |
| family | Family of the species as per Amphibian Species of the World database. |
| subfamily | Subfamily of the species as per Amphibian Species of the World database. |
| genera | Genera of the species as per Amphibian Species of the World database. |
| species | Species as per Amphibian Species of the World database. |
| iucn | Red list conservation status. |
| country | Country name. |
| iso2 | Country code as per ISO 3166-1 alpha-2 standard. |
| iso3 | Country code as per ISO 3166-1 alpha-3 standard. |
| geographic_area | The geographic area that asigned to a IUCN work group. |
| study_yr_first | First year of study. |
| study_yr_last | Last year of study. |
| sample_yr_first | The year when the species was sampled for the first time in the study. |
| sample_yr_last | The year when the species was sampled for the last time in the study. |
| inf_yr_first | The year when the species was first detected as infected in the study. |
| inf_yr_last | The year when the species was last detected as infected in the study. |
| chytrid | If a test for *Bd* turned positive or not. |
| museum | Whether or not the species came from a collection or museum. |

**Supplementary Materials Table 2**

A comma separated value table containing summarized data of infection per country.

The rows are ordered in descending order to show the countries with more species on top.

The symbol “-” is used for missing data to avoid confusions with the iso2 code of Namibia (NA).

| **Field name** | **Description** |
| --- | --- |
| iso3 | Country code as per ISO 3166-1 alpha-3 standard. |
| iso2 | Country code as per ISO 3166-1 alpha-2 standard. |
| geographic_area | The geographic area that assigned to a IUCN work group. |
| last_study_year | The latest year an amphibian was sampled in the country appearing in the data. |
| paper_num | Number of papers from that country in our study. |
| sp_num | Number of amphibian species in the country (according to IUCN). |
| sp_sampled | Number of species in the country that appear in the study. |
| sp_bd_inf | Number of species that tested positive for *Bd* infection in the country. |
| EN | Number of species in the country with IUCN Endangered status. |
| LC | Number of species in the country with IUCN Least Concern status. |
| NT | Number of species in the country with IUCN Near Threatened status. |
| CR | Number of species in the country with IUCN Critical status. |
| VU | Number of species in the country with IUCN Vulnerable status. |
| DD | Number of species in the country with IUCN Data Deficient status. |
| EX | Number of species in the country with IUCN Extinct status (including extinct in the wild). |

**Supplementary Materials Table 3**

A comma separated value table containing summarized data of infection per amphibian family.

The rows are ordered in descending order to show the amphibian families with more species on top.

| **Field name** | **Description** |
| --- | --- |
| family | Amphibian family. |
| sp_num | Number of amphibian species in the amphibian family (according to IUCN). |
| sp_sampled | Number of species that in the amphibian family that appear in the study. |
| sp_bd_inf | Number of species that tested positive for *Bd* infection in the amphibian family. |
| EN | Number of species in the country with IUCN Endangered status. |
| LC | Number of species in the country with IUCN Least Concern status. |
| NT | Number of species in the country with IUCN Near Threatened status. |
| CR | Number of species in the country with IUCN Critical status. |
| VU | Number of species in the country with IUCN Vulnerable status. |
| DD | Number of species in the country with IUCN Data Deficient status. |

**Supplementary Materials Table 4**

A comma separated value table containing first date of *Bd* infection.

The symbol “-” is used for missing data to avoid conflict with the iso2 code of Namibia (NA).

The rows are ordered in descending order to show the countries with more species on top.

| **Field name** | **Description** |
| --- | --- |
| iso2 | Country code as per ISO 3166-1 alpha-2 standard. |
| iso3 | Country code as per ISO 3166-1 alpha-3 standard. |
| possible_first_detection | Possible first detection. Often given when infection is reported but no exact date is provided. |
| first_detection | Year when infection was first reported with an exact date. |
| wos_id | Web of Science accession number of the paper reporting first detection. |
| reference | Bibliography referencing the document “Supplement Materials Bibliography”. |

**Supplementary Materials Table 5**

A list of the Web of Science accession numbers that our query returned. All these papers have been scanned and don’t need to be checked again in future reviews updating this analysis.
