# Supplementary material for: Tracking Batrachochytrium dendrobatidis Infection Across the Globe
Source: Ecohealth. 2020 Nov 17;17(3):270–9. doi: 10.1007/s10393-020-01504-w (PMC7719156; doi:10.1007/s10393-020-01504-w)
Supplement: Supplementary file 2 — Supplementary material 2 (PDF 297 kb) [file 10393_2020_1504_MOESM2_ESM.pdf]

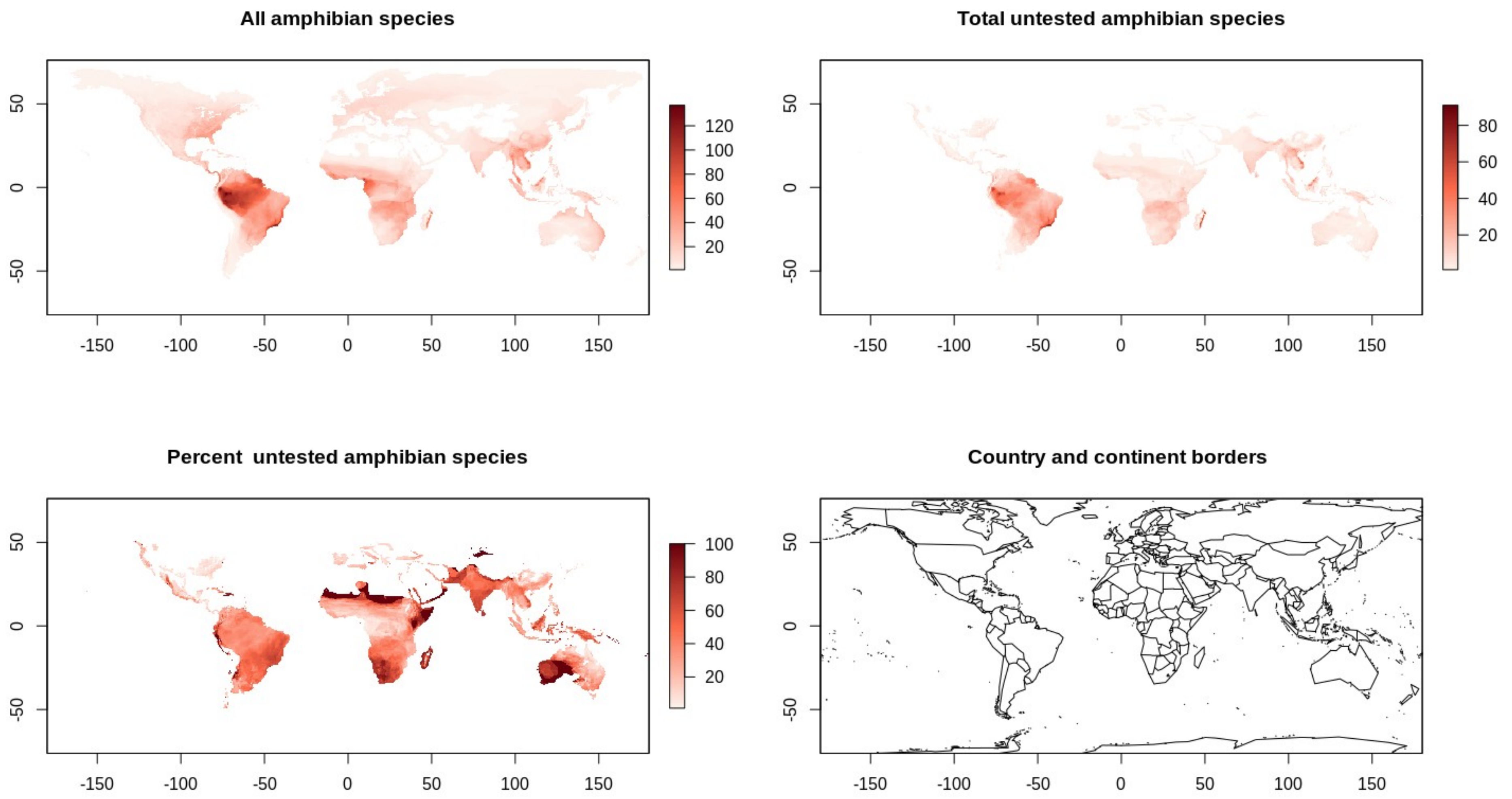

Supplementary Figure 1

The top left plot displays the distribution of amphibians over the world. Notice a great concentration of amphibians species in the equatorial regions, particularly in South America, Central Africa and Southeast Asia. These regions were not equally sampled (top right plot). This is further highlighted by the percentage of untested amphibians (bottom left). Country and continent borders were not overlayed, as they might hinder visualization of coastal/border areas of interest (e.g. Madagascar and Central America). They are displayed for reference on the bottom right plot.

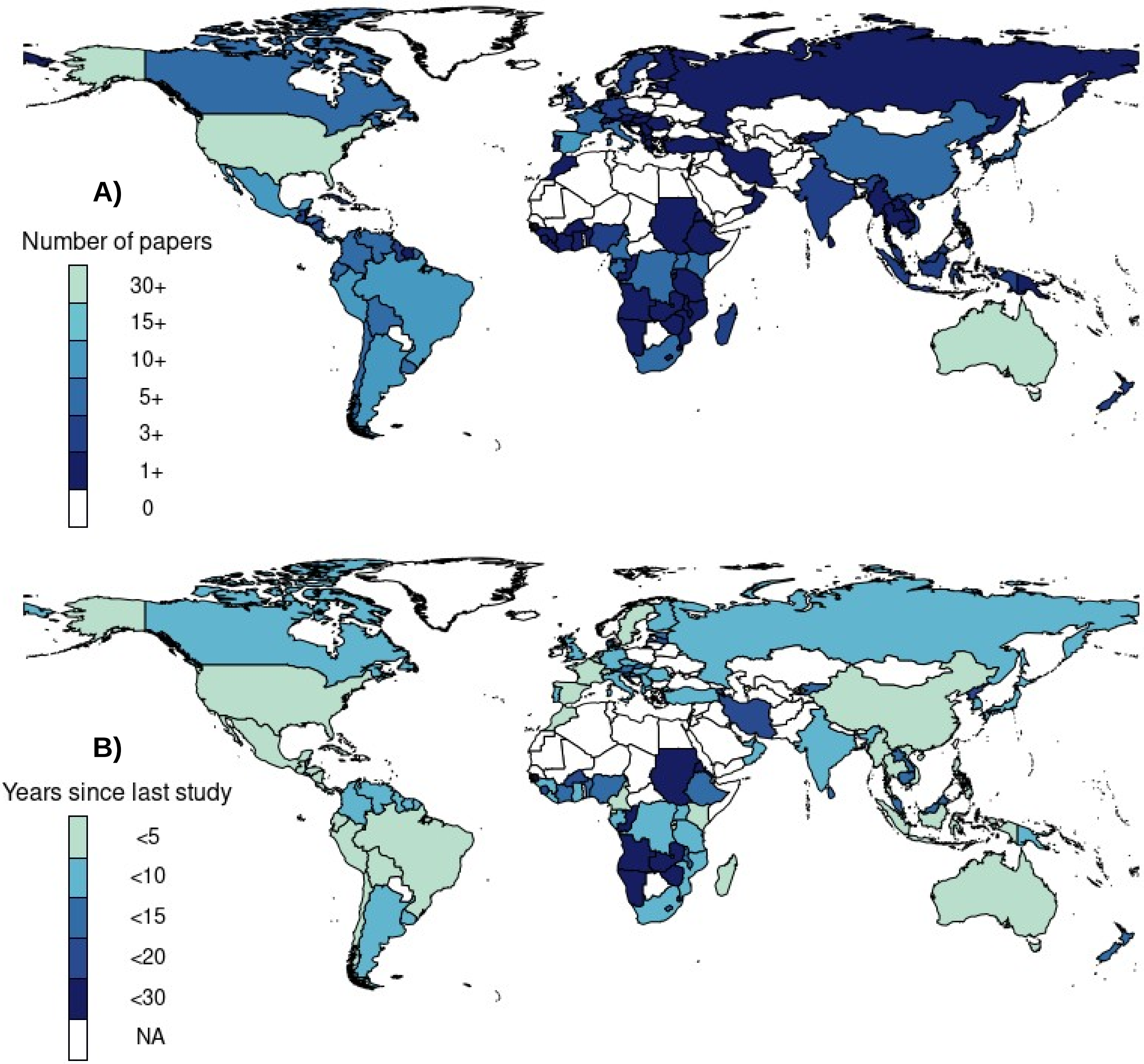

Supplementary Figure 2
There are strong differences in research intensity among countries. Countries for which **A)** only few publications appear in our dataset or **B)** a long time has passed since the most recent paper was published are displayed in dark blue. Notice also countries in white do not appear in any of the 554 publications used in this study. **A)** While most countries have been explored by less than 10 papers, amphibians in the USA have been studied in 198 papers. **B)** Some countries are seldom studied and data from these countries might have been collected several years ago. New recent studies from these areas are much needed. *For specific data points of all countries in the dataset see Supplementary Table 2.*
