# Supplementary material for: Tracking Batrachochytrium dendrobatidis Infection Across the Globe
Source: Ecohealth. 2020 Nov 17;17(3):270–9. doi: 10.1007/s10393-020-01504-w (PMC7719156; doi:10.1007/s10393-020-01504-w)
Supplement: Supplementary file 3 — Supplementary material 3 (PDF 186 kb) [file 10393_2020_1504_MOESM3_ESM.pdf]

Sources Bibliography

Acevedo AA, Martínez MP, Armesto LO, Florez LS, Pérez KS, Lizcano DJ (2016) Detection of *Batrachochytrium dendrobatidis* in amphibians from Northeastern Colombia. *Herpetological Review* 47:220-226

Adams AJ, Pessier AP, Briggs CJ (2017) Rapid extirpation of a North american frog coincides with an increase in fungal pathogen prevalence: Historical analysis and implications for reintroduction. *Ecology and Evolution* 7:10216-10232

Adams MJ, Chelgren ND, Reinitz D, Cole RA, Rachowicz LJ, Galvan S, McCreary B, Pearl CA, Bailey LL, Bettaso J, Bull EL, Leu M (2010) Using occupancy models to understand the distribution of an amphibian pathogen *Batrachochytrium dendrobatidis*. *Ecological Applications* 20:289-302

Adams AJ, Kupferberg SJ, Wilber MQ, Pessier AP, Grefsrud M, Bobzien S, Vredenburg V T, Briggs CJ (2017) Extreme drought host density sex and bullfrogs influence fungal pathogen infection in a declining lotic amphibian. *Ecosphere* 8 e01740

Alemu JB, I, Cazabon MNE, Dempewolf L, Hailey A, Lehtinen RM, Mannette RP, Naranjit KT, Roach ACJ (2008) Presence of the chytrid fungus *Batrachochytrium dendrobatidis* in populations of the critically endangered frog *Mannophryne olmonae* in Tobago West Indies. *EcoHealth* 5:34-39

An D, Waldman B (2016) Enhanced call effort in Japanese tree frogs infected by amphibian chytrid fungus. *Biology Letters* 1220160018

Araos HL, Kroft KL, Bogardus RM, Chang YM, Donohue KR, Hanley D, Hatch KA, Wilson KW (2017) The columbia spotted frog (*Rana luteiventris*)— another species persisting with *Batrachochytrium dendrobatidis* infection. *Herpetol Rev* 47:782-786

Arellano LM, Ferraro DP, Steciow MM, Lavilla EO (2009) Infection by the chytrid fungus *Batrachochytrium dendrobatidis* in the yellow belly frog (*Elachistocleis bicolor*) from Argentina. *Herpetological Journal* 19:217-220

Arellano ML, Akmentins MS, Velasco MA, Kass C, Kacoliris FP (2015) First report of *Batrachochytrium dendrobatidis* in *Atelognathus reverberii* a threatened species in Argentina. *Herpetological Review* 46:354-356

Arellano ML, Velasco MA, Kacoliris FP, Belasen AM, James TY (2017) First record of *Batrachochytrium dendrobatidis* in *Pleurodema somuncurense*, a critically endangered species from Argentina. *Herpetological Review* 48:68–70

Ayres C, Acevedo I, Monsalve-Carcaño C, Thumsová B, Bosch J (2020) Triple dermocystid-chytrid fungus-*Ranavirus* co-infection in a *Lissotriton helveticus*. *European Journal of Wildlife Research* 66 https://doi.org/10.1007/s10344-020-01381-2

Aziz MNBA, Skerratt LF, Mc Callum H. (2011) Dynamics of chytridiomycosis in a Tasmanian frog community. *Herpetological Review* 42:53-57

Azmanis PN, Strachinis I, Lymberakis P, Marschang RE (2016) First detection of the amphibian chytrid fungus (Batrachochytridium *dendrobatidis*) in free-living anuran populations in Greece. *Journal of the Hellenic Veterinary Medical Society* 67:253-258

Bacigalupe LD, Soto-Azat C, García-Vera C, Barría-Oyarzo I, Rezende EL (2017) Effects of amphibian phylogeny climate and human impact on the occurrence of the amphibian-killing chytrid fungus. *Global Change Biology* 23:3543-3553

Backlin AR, Hitchcock CJ, Gallegos EA, Yee JL, Fisher RN (2015) The precarious persistence of the endangered sierra madre yellow-legged frog *rana muscosa* in Southern California, USA. *Oryx* 49:157-164

Bai C, Garner TWJ, Li Y (2010) First evidence of *Batrachochytrium dendrobatidis* in China: Discovery of chytridiomycosis in introduced american bullfrogs and native amphibians in the Yunnan province, China. *EcoHealth* 7:127-134

Bai C, Liu X, Fisher MC, Garner TWJ, Li Y (2012) Global and endemic Asian lineages of the emerging pathogenic fungus *Batrachochytrium dendrobatidis* widely infect amphibians in China. *Diversity and Distributions* 18:307-318

Balaz V, Kopecky O, Gvozdik V (2012) Presence of the amphibian chytrid pathogen confirmed in Cameroon. *Herpetological Journal* 22:191-194

Balaz V, Vörös J, Civis P, Vojar J, Hettyey A, Sos E, Dankovics R, Jehle R, Christiansen DG, Clare F, Fisher MC, Garner TWJ, Bielby J (2014a) Assessing risk and guidance on monitoring of *Batrachochytrium dendrobatidis* in Europe through identification of taxonomic selectivity of infection. *Conservation Biology* 28:213-223

Balaz V, Vojar J, Civis P, Sandera M, Rozinek R (2014b) Chytridiomycosis risk among Central European amphibians based on surveillance data. *Diseases of Aquatic Organisms* 112:1-8

Bales EK, Hyman OJ, Loudon AH, Harris RN, Lipps G, Chapman E, Roblee K, Kleopfer JD, Terrell KA (2015) Pathogenic chytrid fungus *Batrachochytrium dendrobatidis*, but not *B. salamandrivorans* detected on Eastern hellbenders. *PLoS ONE* 10 e0116405

Barber D (2012) Chytrid Fungus *Batrachochytrium dendrobatidis* (*Bd*) detected at lower elevations in Puerto Rico: Implications for conservation of Puerto Rican crested toad (*Peltophryne lemur*). *Herpetological Review* 43:73-75

Bardier C, Ghirardi R, Levy M, Maneyro R (2011) First case of chytridiomycosis in an adult specimen of a native anuran from Uruguay. *Herpetological Review* 42:65-66

Barrera GS, García AP (2018) Earliest Record of *Batrachochytrium dendrobatidis* in amphibian populations of Baja California, Mexico. *Herpetological Review* 49:693–695

Barrionuevo JS, Aguayo R, Lavilla EO (2008) First record of chytridiomycosis in Bolivia (*Rhinella quechua*; Anura: Bufonidae). *Diseases of Aquatic Organisms* 82:161-163

Barrionuevo S, Mangione S (2006) Chytridiomycosis in two species of Telmatobius (Anura : Leptodactylidae) from Argentina. *Diseases of Aquatic Organisms* 73:171-174

Basanta DM, Calzada-arciniega RA, Velázquez GJ, Arias-balderas SF, Reyes AAI, Medina Rangel G, Suazo-Ortuño I, Ochoa-Ochoa LM, Parra-Olea G (2019) Detection of *Batrachochytrium dendrobatidis* in threatened endemic mole salamanders (Ambystoma) in Mexico. *Herpetological Review* 50 493–495

Bataille A, Fong JJ, Cha M, Wogan GOU, Baek HJ, Lee H, Min M-S, Waldman B (2013) Genetic evidence for a high diversity and wide distribution of endemic strains of the pathogenic chytrid fungus *Batrachochytrium dendrobatidis* in wild Asian amphibians. *Molecular Ecology* 22:4196-4209

Bates KA, Clare FC, O'Hanlon S, Bosch J, Brookes L, Hopkins K, McLaughlin EJ, Daniel O, Garner TWJ, Fisher MC, Harrison XA (2018) Amphibian chytridiomycosis outbreak dynamics are linked with host skin bacterial community structure. *Nature Communications* 9 693

Battaglin WA, Smalling KL, anderson C, Calhoun D, Chestnut T, Muths E (2016) Potential interactions among disease pesticides water quality and adjacent land cover in amphibian habitats in the united states. *Science of the Total Environment* 566:320-332

Beard K, O'Neill E (2005) Infection of an invasive frog *Eleutherodactylus coqui* by the chytrid fungus *Batrachochytrium dendrobatidis* in Hawaii. *Biological Conservation* 126:591-595

Becker CG, Rodriguez D, Longo AV, Talaba AL, Zamudio KR (2012) Disease risk in temperate amphibian populations is higher at closed-canopy sites. *PLoS ONE* 7 e48205

Becker CG, Longo AV, Haddad CFB, Zamudio KR (2017) Land cover and forest connectivity alter the interactions among host pathogen and skin microbiome. *Proceedings of the Royal Society B: Biological Sciences* 284 20170582

Becker CG, Bletz MC, Greenspan SE, Rodriguez D, Lambertini C, Jenkinson TS, Guimaraes PR, Assis APA, Geffers R, Jarek M, Toledo LF, Vences M, Haddad CFB (2019) Low-load pathogen spillover predicts shifts in skin microbiome and survival of a terrestrial-breeding amphibian. *Proceedings of the Royal Society B: Biological Sciences* 286 (1908) 20191114

Bell RC, Garcia AVG, Stuart BL, Zamudio KR (2011) High prevalence of the amphibian chytrid pathogen in Gabon. *EcoHealth* 8:116-120

Bell SC, Garland S, Alford RA (2018) Increased numbers of culturable inhibitory bacterial taxa may mitigate the effects of *Batrachochytrium dendrobatidis* in Australian wet tropics frogs. *Frontiers in Microbiology* 9

Benício RA, Carvalho T, Barbosa MDR, Costa J de M, da Silva FCC, Fonseca MG (2019) Worrying news for Brazilian Caatinga: Prevalence of *Batrachochytrium dendrobatidis* in amphibians. *Tropical Conservation Science* 12 194008291989262

Berenguel RA, Elias RK, Weaver TJ, Reading RP (2016) Chytrid fungus *Batrachochytrium dendrobatidis* in wild populations of the Lake Titicaca frog telmatobius culeus in Peru. *Journal of Wildlife Diseases* 52:973-975

Berger L, Speare R, Daszak P, Green D, Cunningham A, Goggin C, Slocombe R, Ragan M, Hyatt A, McDonald K, Hines H, Lips K, Marantelli G, Parkes H (1998) Chytridiomycosis causes amphibian mortality associated with population declines in the rain forests of Australia and Central America. *Proceedings of the National Academy of Sciences of the United States of America.* 95:9031-9036

Berger L, Speare R, Hines H, Marantelli G, Hyatt A, McDonald K, Skerratt L, Olsen V, Clarke J, Gillespie G, Mahony M, Sheppard N, Williams C, Tyler M (2004) Effect of season and temperature on mortality in amphibians dueto chytridiomycosis. *Australian Veterinary Journal* 82:434-439

Berger L, Speare R, Skerratt L (2005) Distribution of *Batrachochytrium dendrobatidis* and pathology in the skin of green tree frogs *Litoria caerulea* with severe chytridiomycosis. *Diseases of Aquatic Organisms* 68:65-70

Beyer SE, Phillips CA, Schooley RL (2015) Canopy cover and drought influence the landscape epidemiology of an amphibian chytrid fungus. *Ecosphere* 6:78

Bielby J, Bovero S, Angelini C, Favelli M, Gazzaniga E, Perkins M, Sotgiu G, Tessa G, Garner TWJ (2013) Geographic and taxonomic variation in *Batrachochytrium dendrobatidis* infection and transmission within a highly endemic amphibian community. *Diversity and Distributions* 19:1153-1163

Bielby J, Bovero S, Sotgiu G, Tessa G, Favelli M, Angelini C, Doglio S, Clare FC, Gazzaniga E, Lapietra F, Garner TWJ (2009) Fatal chytridiomycosis in the Tyrrhenian painted frog. *EcoHealth* 6:27-32

Bishop PJ, Speare R, Poulter R, Butler M, Speare BJ, Hyatt A, Olsen V, Haigh A (2009) Elimination of the amphibian chytrid fungus *Batrachochytrium dendrobatidis* by Archey's frog *Leiopelma archeyi*. *Diseases of Aquatic Organisms* 84:9-15

Bakkegard KA, Pessier AP (2010) *Batrachochytrium dendrobatidis* in adult *Notophthalmus viridescens* in North-Central Alabama, USA. *Herpetological Review* 41:45-47

Blackburn M, Wayland J, Smith WH, McKenna JH, Harry M, Hamed MK, Gray MJ, Miller DL (2015) First report of *Ranavirus* and *Batrachochytrium dendrobatidis* in green salamanders (*Aneides aeneus*) from Virgina, USA. *Herpetological Review* 46:357-361

Bletz MC, Harris RN (2013) Occurrence of *Batrachochytrium dendrobatidis* in *Notophthalmus viridescens* in Northwestern Virginia, USA. *Herpetological Review* 44:257-259

Bletz MC, Rosa GM, andreone F, Courtois EA, Schmeller DS, Rabibisoa NHC, Rabemananjara FCE, Raharivololoniaina L, Vences M, Weldon C, Edmonds D, Raxworthy CJ, Harris RN, Fisher

MC, Crottini A (2015a) Widespread presence of the pathogenic fungus *Batrachochytrium dendrobatidis* in wild amphibian communities in Madagascar. *Scientific Reports* 5:8633

Bletz MC, Rosa GM, andreone F, Courtois EA, Schmeller DS, Rabibisoa NHC, Rabemananjara FCE, Raharivololoniaina L, Vences M, Weldon C, Edmonds D, Raxworthy CJ, Harris RN, Fisher MC, Crottini A (2015b) Consistency of published results on the pathogen *Batrachochytrium dendrobatidis* in madagascar: Formal comment on Kolby et al. Rapid response to evaluate the presence of amphibian chytrid fungus (*Batrachochytrium dendrobatidis*) and *Ranavirus* in wild amphibian populations in Madagascar. *PLoS ONE* 10 e0134524

Bletz MC, Vences M, Sabino-Pinto J, Taguchi Y, Shimizu N, Nishikawa K, Kurabayashi A (2017) Cutaneous microbiota of the Japanese giant salamander (*Andrias japonicus*) a representative of an ancient amphibian clade. *Hydrobiologia* 795:153-167

Blooi M, Laking AE, Martel A, Haesebrouck F, Jocque M, Brown T, Green S, Vences M, Bletz, MC, Pasmans F (2017) Host niche may determine disease-driven extinction risk. *PLOS ONE* 12 e0181051

Bodinof CM, Briggler JT, Duncan MC, Beringer J, Millspaugh JJ (2011) Historic occurrence of the amphibian chytrid fungus *Batrachochytrium dendrobatidis* in hellbender *Cryptobranchus alleganiensis* populations from Missouri. *Diseases of Aquatic Organisms* 96:1-7

Boivin E (2012) *Batrachochytrium dendrobatidis* in the Adirondacks New York, USA. *Herpetological Review* 43:610

Bonaccorso E, Guayasamin JM, Mendez D, Speare R (2003) Chytridiomycosis as a possible cause of population declines in *Atelopus cruciger* (Anura: Bufonidae). *Herpetological Review* 34:331-334

Borteiro C, Carlos Cruz J, Kolenc F, Aramburu A (2009) Chytridiomycosis in frogs from Uruguay. *Diseases of Aquatic Organisms* 84:159-162

Borteiro C, Carlos Cruz J, Kolenc F, Manuel Verdes J, Morana A, Martinez Debat C, Kun A, Ubilla M, Okada K (2014) Dermocystid-chytrid coinfection in the neotropical frog *Hypsiboas pulchellus* (Anura: Hylidae). *Journal of Wildlife Diseases* 50:150-153

Borteiro C, Kolenc F, Verdes JM, Martínez Debat C, Ubilla M (2019) Sensitivity of histology for the detection of the amphibian chytrid fungus *Batrachochytrium dendrobatidis. Journal of Veterinary Diagnostic Investigation* 31:246-249

Borzée A, Kosch TA, Kim M, Jang Y (2017) Introduced bullfrogs are associated with increased *Batrachochytrium dendrobatidis* prevalence and reduced occurrence of Korean treefrogs. *PLOS ONE* 12 e0177860

Bosch J, Martinez-Solano I, Garcia-Paris M (2001) Evidence of a chytrid fungus infection involved in the decline of the common midwife toad (*Alytes obstetricans*) in protected areas of Central Spain. *Biological Conservation* 97:331-337

Bosch J, Martinez-Solano I (2006) Chytrid fungus infection related to unusual mortalities of *Salamandra salamandra* and *Bufo bufo* in the Peñalara Natural Park, Spain. *Oryx* 40:84-89

Bosch J, Garcia-Alonso D, Fernandez-Beaskoetxea S, Fisher MC, Garner TWJ (2013) Evidence for the introduction of lethal chytridiomycosis affecting wild betic midwife toads (*Alytes dickhilleni*). *EcoHealth* 10:82-89

Bosch RA, García Padrón LY, Almaguer M, Valle M (2017) First reports of tadpole mouthpart anomalies in a Cuban toad (Anura: Bufonidae: Peltophryne). *Herpetological Review* 48:58–62.

Bosch J, Fernández-Beaskoetxea S, Garner TWJ, Carrascal LM (2018) Long-term monitoring of an amphibian community after a climate change- and infectious disease-driven species extirpation. *Global Change Biology* 24:2622-2632

Bourke J, Mutschmann F, Ohst T, Ulmer P, Gutsche A, Busse K, Werning H, Boehme W (2010) *Batrachochytrium dendrobatidis* in Darwin's frog *Rhinoderma Spp*. in Chile. *Diseases of Aquatic Organisms* 92:217-221

Bourke J, Ohst T, Graeser Y, Boehme W, Plötner J (2011) New records of *Batrachochytrium dendrobatidis* in chilean frogs. *Diseases of Aquatic Organisms* 95:259-261

Bovero S, Sotgiu G, Angelini C, Doglio S, Gazzaniga E, Cunningham AA, Garner TWJ (2008) Detection of chytridiomycosis caused by *Batrachochytrium dendrobatidis* in the endangered sardinian newt (*Euproctus platycephalus*) in Southern Sardinia Italy. *Journal of Wildlife Diseases* 44:712-715

Böll S, Tobler U, Geiger CC, Hansbauer G, Schmidt BR (2012) The amphibian chytrid fungus in Bavarian populations of *Alytes obstetricans*: Past absence current presence and metamorph mortality. *Amphibia-Reptilia* 33:319-326

Bradley G, Rosen P, Sredl M, Jones T, Longcore J (2002) Chytridiomycosis in native Arizona frogs. *Journal of Wildlife Diseases* 38:206-212

Brannelly LA, Hunter DA, Lenger D, Scheele BC, Skerratt LF, Berger L (2015) Dynamics of chytridiomycosis during the breeding season in an Australian alpine amphibian. *PLoS ONE* 10 e0143629

Brannelly LA, Chatfield MWH, Sonn J, Robak M, Richards-Zawacki CL (2018) Fungal infection has sublethal effects in a lowland subtropical amphibian population. BMC Ecology 18

Brem FMR, Lips KR (2008) *Batrachochytrium dendrobatidis* Infection patterns among panamanian amphibian species habitats and elevations during epizootic and enzootic. *Diseases of Aquatic Organisms* 81:189-202

Brannelly LA, Webb RJ, Hunter DA, Clemann N, Howard K, Skerratt LF, Berger L, Scheele BC (2017) Non-declining amphibians can be important reservoir hosts for amphibian chytrid fungus. *Animal Conservation* 21:91-101

Bresciano JC, Salvador CA, Paz-y-Mino C, Parody-Merino AM, Bosch J, Woodhams DC (2015) Variation in the presence of Anti-*Batrachochytrium dendrobatidis* Bacteria of amphibians across life stages and elevations in Ecuador. *EcoHealth* 12:310-319

Briggs C, Burgin S. 2003. A Rapid Technique to Detect Chytrid Infection in Adult Frogs. *Herpetological Review* 34:124-126

Briggs CJ, Knapp RA, Vredenburg VT (2010) Enzootic and epizootic dynamics of the chytrid fungal pathogen of amphibians. *Proceedings of the National Academy of Sciences of the United States of America.* 107:9695-9700

Brown J, Kerbby J (2013) *Batrachochytrium dendrobatidis* in South Dakota, USA amphibians. *Herpetological Review* 44:457-458

Bull EL (2006) Sexual differences in the ecology and habitat selection of Western toads (*Bufo boreas*) in Northeastern Oregon. *Herpetological Conservation and Biology* 1:27-38

Burgmeier NG, Unger SD, Meyer JL, Sutton TM, Williams RN (2011) Health and habitat quality assessment for the Eastern hellbender (*Cryptobranchus alleganiensis alleganiensis*) in Indiana, USA. *Journal of Wildlife Diseases* 47:836-848

Burrowes P, Joglar R, Green D (2004) Potential causes for amphibian declines in Puerto Rico. *Herpetologica* 60:141-154

Burrowes P, Longo AV, Joglar RL, Cunningham AA (2008) Geographic distribution of *Batrachochytrium dendrobatidis* in Puerto Rico. *Herpetological Review* 39:321-324

Burrowes PA, Alicea A, Longo AV, Joglar RL (2011) Toes versus swabs? Evaluation of the best tissue source for detection of *Batrachochytrium dendrobatidis* in field-caught amphibians. *Herpetological Review* 42:359-362

Burrowes PA, De la Riva I (2017) Unraveling the historical prevalence of the invasive chytrid fungus in the Bolivian Andes: Implications in recent amphibian declines. *Biological Invasions* 19:1781-1794

Burrowes PA, Martes MC, Torres-Ríos M, Longo AV (2017) Arboreality predicts *Batrachochytrium dendrobatidis* infection level in tropical direct-developing frogs. *Journal of Natural History* 51:643-656

Byrne AQ, Vredenburg VT, Martel A, Pasmans F, Bell RC, Blackburn DC, Bletz MC, Bosch J, Briggs CJ, Brown RM, Catenazzi A, Familiar López M, Figueroa-Valenzuela R, Ghose SL, Jaeger JR, Jani AJ, Jirku M, Knapp RA, Muñoz A, Portik DM, Richards-Zawacki CL, Rockney H, Rovito SM, Stark T, Sulaeman H, Tao NT, Voyles J, Waddle AW, Yuan ZY, Rosenblum EB (2019) Cryptic diversity of a widespread global pathogen reveals expanded threats to amphibian conservation. *Proceedings of the National Academy of Sciences* 116:20382-20387

Byrne MW, Davie EP, Gibbons JW (2008) *Batrachochytrium dendrobatidis* occurrence in *Eurycea cirrigera*. South*eastern Naturalist* 7:551-555

Carey C, Livo LJ (2009) Chytridiomycosis in Woodhouse's toad (*Anaxyrus woodhousii*) in Colorado. *Herpetological Review* 40:52-54

Cádiz A, Reytor ML, Díaz LM, Chestnut T, Burns JA, Amato G (2018) The chytrid fungus *Batrachochytrium dendrobatidis* is widespread among Cuban amphibians. *Ecohealth* 16:128-140

Canestrelli D, Zampiglia M, Nascetti G (2013) Widespread occurrence of *Batrachochytrium dendrobatidis* in contemporary and historical samples of the endangered *Bombina pachypus* along the Italian Peninsula. *PLoS ONE* 8 e63349

Caruso NM, Lips KR (2013) Truly enigmatic declines in terrestrial salamander populations in Great Smoky Mountains National Park. *Diversity and Distributions* 19:38-48

Cashins SD, Philips A, Skerratt LF (2015) Using site-occupancy models to prepare for the spread of chytridiomyosis and identify factors affecting detectability of a cryptic susceptible species the Tasmanian tree frog. *Wildlife Research* 42:405-413

Catenazzi A, Lehr E, Rodriguez LO, Vredenburg VT (2011) *Batrachochytrium dendrobatidis* and the collapse of anuran species richness and abundance in the Upper Manu National Park Southeastern Peru. *Conservation Biology* 25:382-391

Catenazzi A, Von May R, Vredenburg VT (2013) High prevalence of infection in tadpoles increases vulnerability to fungal pathogen in high-andean amphibians. *Biological Conservation* 159:413-421

Catenazzi A, Lehr E, Vredenburg VT (2014) Thermal physiology, disease, and amphibian declines diversity and distributions on the Eastern slopes of the Andes. *Conservation Biology* 28:509-517

Catenazzi A, Swei A, Finkle J, Foreyt E, Wyman L, Vredenburg VT (2017) Epizootic to enzootic transition of a fungal disease in tropical andean frogs: Are surviving species still susceptible? *PLOS ONE* 12 e0186478

Catullo RA, Morgan MJ, Piggott MP, Alford RA (2018) *Batrachochytrium dendrobatidis* surveys from the Savannah Regions of Northern and Central Queensland, Australia. *Herpetological Review* 49:41–44

Chaber A-L, Combreau O, Perkins M, SaeGerman C, Cunningham A (2016) Preliminary surveys fail to detect *Batrachochytrium dendrobatidis* infection in the United Arab Emirates and Oman. *Herpetological Review* 47:403-404

Chatfield MWH, Moler P, Richards-Zawacki CL (2012) The amphibian chytrid fungus *Batrachochytrium dendrobatidis* in fully aquatic salamanders from Southeastern North America. *PLoS ONE* 7 e44821

Chiari Y, van der Meijden A, Mucedda M, Wagner N, Veith, M (2013) No detection of the pathogen *Batrachochytrium dendrobatidis* in sardinian cave salamanders, genus hydromantes. *Amphibia-Reptilia* 34:136-141

Chiari Y, Moreno N, Elmore J, Hylton A, Ray A, Burkhardt A, Glaberman S (2017) Widespread occurrence of *Batrachochytrium dendrobatidis* in Southern Alabama, USA. *Herpetological Review* 48:356–359

Cheng TL, Rovito SM, Wake DB, Vredenburg VT (2011) Coincident mass extirpation of neotropical amphibians with the emergence of the infectious fungal pathogen *Batrachochytrium dendrobatidis*. *Proceedings of the National Academy of Sciences of the United States of America*. 108:9502-9507

Chestnut T, Johnson JE, Wagner SR, 2008:Results of amphibian chytrid *(Batrachochytrium dendrobatidis)* sampling in Denali National Park Alaska, USA. *Herpetological Review* 39: 202-204

Chong SM, Sng W, Yan BTZ, Wong WK, Siow HJ, Fernandez CJ (2018) Prevalence of chytrid fungus *Batrachochytrium dendrobatidis* in wild amphibians, Singapore. *Herpetological Review* 49:252–254

Civiš, P, Vojar J, Literák I, Baláž, V (2012) Current state of *Bd* occurrence in the Czech Republic. *Herpetological Review* 43:75-78

Clare F, Daniel O, Garner T, Fisher M (2016) Assessing the ability of swab data to determine the true burden of infection for the amphibian pathogen *Batrachochytrium dendrobatidis*. *EcoHealth* 13:360-367

Conradie W, Weldon C, Smith KG, Du Preez LH (2011) Seasonal pattern of chytridiomycosis in common river frog (*Amietia angolensis*) tadpoles in the South African grassland biome. *African Zoology* 46:95-102

Conradie W, Harvey J, Kotzé, A, Dalton DL, Cunningham MJ (2011b) Confirmed amphibian chytrid in Mount Mulanje area Malawi. *Herpetological Review* 42:369-371

Conradie W, Bittencourt-Silva GB, Loader SP, Menegon M, Nanvonamuquitxo C, Kotzè, A, Dalton DL, Engelbrecht HM, Tolley KA (2016) *Batrachochytrium dendrobatidis* Survey of amphibians in the Northern mozambique "sky islands" and low-lying areas. *Herpetological Review* 47:42-46

Cook K, Voyles J, Kenny H, Pope K, Piovia-Scott J (2018) Non-lethal isolation of the fungal pathogen *Batrachochytrium dendrobatidis* (*Bd*) from amphibians. *Diseases of Aquatic Organisms* 129:159-164

Cossel J, Jr Lindquist E, Craig H, Luthman K (2014) Pathogenic fungus *Batrachochytrium dendrobatidis* in marbled water frog *Telmatobius marmoratus*: First record from Lake Titicaca Bolivia. *Diseases of Aquatic Organisms* 112:83-87

Courtois EA, Gaucher P, Chave J, Schmeller DS (2015) Widespread occurrence of *Bd* in French Guiana, South America. *PLoS ONE* 10 e0125128

Crespi EJ, Rissler LJ, Mattheus NM, Engbrecht K, Duncan SI, Seaborn T, Hall EM, Peterson JD, Brunner JL (2015) Geophysiology of Wood Frogs: Landscape Patterns of Prevalence of Disease and Circulating Hormone Concentrations Across the Eastern Range. *Integrative and Comparative Biology* 55:602-617

Crottini A, Barbuto M, Casiraghi M, Andreone F (2011) A Rapid Amphibian Survey at Itremo-Ambatofinandrahana, Central Madagascar, with Confirmed Absence of Chytrid Fungus and Recommendations for Future Monitoring Activities. North *Western Journal of Zoology* 7, 346-351

Crottini A, Bollen A, Weldon C, Dalton DL, Kotze A, Noel J, Iambana B, Andreone F (2014) Amphibian survey and current absence of *Batrachochytrium dendrobatidis* (*Bd*) in Ivoloina Park, Toamasina (Eastern Madagascar). *African Journal of Herpetology* 63:70-78

Cummer MR, Green ED, O'neil EM (2005) Aquatic chytrid pathogen detected in terrestrial plethodontid salamander. *Herpetological Review* 36:248-249

Cusi JC, Barboza AC, Vredenburg VT, von May R (2017) New distribution records and conservation status of *Atelopus seminiferus cope*, 1874: A critically endangered harlequin frog from Northern Peru. Amphibian, Reptile Conservation 11:17-24

Dahanukar N, Krutha K, Paingankar MS, Padhye AD, Modak N, Molur S (2013) Endemic Asian chytrid strain infection in threatened and endemic anurans of the Northern Western Ghats, India. *PLoS ONE* 8 e77528

D'Aoust-Messier A-M, Echaubard P, Billy V, Lesbarreres D (2015) Amphibian pathogens at Northern latitudes: Presence of chytrid fungus and *Ranavirus* in Northeastern Canada. *Diseases of Aquatic Organisms* 113:149-155

Daszak P, Scott D, Kilpatrick A, Faggioni C, Gibbons J, Porter D (2005) Amphibian population declines at Savannah River site are linked to climate not chytridiomycosis. *Ecology* 86:3232-3237

Daversa D, Bosch J, Jeffrey K (2011) First survey of the chytrid fungus *Batrachochytrium dendrobatidis* in amphibian populations of Gabon Africa. *Herpetological Review* 42:67-69

Daversa DR, Monsalve-Carcaño C, Carrascal LM, Bosch J (2018) Seasonal migrations body temperature fluctuations and infection dynamics in adult amphibians. *PeerJ* 6 e4698

Davidson S. R. A, Chambers DL (2011) Occurrence of *Batrachochytrium dendrobatidis* in amphibians of Wise County, Virginia, USA. *Herpetological Review* 42:214-215

Davis JR, Eastlack DT, Kouba AJ, Vance CK (2012) *Batrachochytrium dendrobatidis* detected in Fowler's toad (*Anaxyrus fowleri*) populations in Memphis Tennessee, USA. *Herpetological Review* 43:81-83

Delgado CS, Natale GS, Herrera RA, Barraso DA (2012) First record of *Batrachochytrium dendrobatidis* in *Physalaemus fernandezae* (Anura: Leiuperidae) for Buenos Aires Province Argentina. *Herpetological Review* 43:84-85

de Oliveira Ramalho AC, De Paula CD, Catao-Dias JL, Vilarinho B, Brandao RA (2013) First record of *Batrachochytrium dendrobatidis* in two endemic cerrado Hylids *Bokermannohyla pseudopseudis* and *Bokermannohyla sapiranga* with comments on chytridiomycosis spreading in Brazil. North-*Western Journal of Zoology* 9:145-150

de La Riva I, Burrowes PA (2011) Rapid assessment of the presence of *Batrachochytrium dendrobatidis* in bolivian andean frogs. *Herpetological Review* 42:372-375

De León ME, Vredenburg VT, Piovia-Scott J (2016) Recent emergence of a chytrid fungal pathogen in california cascades frogs (*Rana cascadae*). *Ecohealth* 14:155-161

Diaz LM, Cadiz A, Chong A, Silva A (2007) First report of chytridiomycosis in a dying toad (Anura : Bufonidae) from Cuba: A new conservation challenge for the island. *EcoHealth* 4:172-175

DiRenzo GV, Campbell Grant EH, Longo AV, Che-Castaldo C, Zamudio KR, Lips KR (2017) Imperfect pathogen detection from non-invasive skin swabs biases disease inference. *Methods in. Ecology and Evolution* 9:380-389

DiRenzo GV, Zipkin EF, Grant EHC, Royle JA, Longo AV, Zamudio KR, Lips KR (2018) Eco-evolutionary rescue promotes host-pathogen coexistence. *Ecological Applications* 28:1948-1962

Doherty-Bone TM, Gonwouo NL, Hirschfeld M, Ohst T, Weldon C, Perkins M, Kouete MT, Browne RK, Loader SP, Gower DJ, Wilkinson MW, Rödel MO, Penner J, Barej MF, Schmitz A, Plötner J, Cunningham AA (2013a) *Batrachochytrium dendrobatidis* in amphibians of Cameroon, including first records for caecilians. *Diseases of Aquatic Organisms* 102:187-194

Doherty-Bone TM, Ndifon RK, Nyingchia ON, Landrie FE, Yonghabi FT, Duffus ALJ, Price S, Perkins M, Bielby J, Kome NB, LeBreton M, Gonwouo LN, Cunningham AA (2013b) Morbidity and mortality of the critically endangered Lake Oku clawed frog *Xenopus longipes*. *Endangered Species Research* 21:115-128

Drake DL, Altig R, Grace JB, Walls SC (2007) Occurrence of oral deformities in larval anurans. *Copeia* 2:449-458
Edwards L, Ross AA, Pike DA (2019) *Batrachochytrium dendrobatidis* In Southern ornate nursery frogs, *Cophixalus australis* (MicroHylidae). *Herpetological Review* 50:288-289
El Cadi RA, Laghzaoui E, Crottini A, Slimani T, Bosch J, El Mouden E (2019) Occurrence of *Batrachochytrium dendrobatidis* in the Tensift region, with comments on its spreading in Morocco. Acta Herpetologica 14:109-115
El Mouden EH, Slimani T, Donaire D, Fernández-Beaskoetxea S, Fisher MC, Bosch J (2011) First record of the chytrid fungus *Batrachochytrium dendrobatidis* in North Africa. *Herpetological Review* 42:71-75
Erdmann JA, Estes-Zumpf WA, Snoberger C, Walker ZJ, Pocewicz A, Kerby JL, Siler CD (2018) Expanding knowledge of *Batrachochytrium dendrobatidis* in Wyoming, USA. *Herpetological Review* 49:37–41
Erismis UC, Konuk M, Yoldas T, Agyar P, Yumuk D, Korcan SE (2014) Survey of Turkey's endemic amphibians for chytrid fungus *Batrachochytrium dendrobatidis*. *Diseases of Aquatic Organisms* 111:153-157
Eskew EA, Todd BD, Hopkins WA (2014) Extremely low prevalence of *Batrachochytrium dendrobatidis* infection in Eastern hellbenders (*Cryptobranchus alleganiensis alleganiensis*) in Southwest Virginia, USA. *Herpetological Review* 45:425-427
Familiar López M, Rebollar EA, Harris RN, Vredenburg VT, Hero J.-M (2017) Temporal variation of the skin bacterial community and *Batrachochytrium dendrobatidis* infection in the terrestrial cryptic frog *Philoria loveridgei*. *Frontiers in Microbiology* 8 2535
Fellers G, Green D, Longcore J (2001) Oral chytridiomycosis in the mountain yellow-legged frog (*Rana muscosa*). *Copeia* 4:945-953
Fellers GM, Cole RA, Reinitz DM, Kleeman PM (2011) Amphibian chytrid fungus (*Batrachochytrium dendrobatidis*) in coastal and montane California, USA anurans. *Herpetological Conservation and Biology* 6:383-394
Fellers GM, Kleeman PM, Miller DAW, Halstead BJ, Link WA (2013) Population size, survival growth, and movements of *Rana sierrae*. *Herpetologica* 69:147-162
Fenolio DB, Charrier A, Levy MG, Farbry MO, Tirado MS, Crump ML, Lamar WW, Calderon P (2011) A review of the Chile mountains false toad *Telmatobufo venustus* (Amphibia: Anura: Calyptocephalellidae) with comments on its conservation status. *Herpetological Review* 42:514-519
Fenolio DB, Moreno-Puig V, Levy M, Lamar WW, Fabry MO, Tirado MS, Crump ML, Charrier A (2013) Status and conservation of a gondwana legacy: Bullock's false toad *Telmatobufo bullocki* (Amphibia: Anura: Calyptocephalellidae). *Herpetological Review* 44:583-590
Fernandez-Beaskoetxea S, Carrascal LM, Fernandez-Loras A, Fisher MC, Bosch J (2015) Short term minimum water temperatures determine levels of infection by the amphibian chytrid fungus in *Alytes obstetricans* Tadpoles. *PLoS ONE* 10 e0120237
Fernández-Loras A, Boyero L, Correa-Araneda F, Tejedo M, Hettyey A, Bosch J (2019) Infection with *Batrachochytrium dendrobatidis* lowers heat tolerance of tadpole hosts and cannot be cleared by brief exposure to CTmax. *PLOS ONE* 14 e0216090
Ferreira TK. Lamarão FR, Moraes MO, Sluys MV (2008) Amphibian chytrid infection in *Melanophryniscus moreirae* (Bufonidae) in the Brazilian atlantic rainforest. *Herpetological Review* 39:445-446
Ficetola GF, Valentini A, Miaud C, Noferini A, Mazzotti S, Dejean T (2011) *Batrachochytrium dendrobatidis* in amphibians from the Po River delta Northern Italy. *Acta Herpetologica* 6:297-302

Flechas SV, Sarmiento C, Cardenas ME, Medina EM, Restrepo S, Amezquita A (2012) Surviving chytridiomycosis: Differential anti-*Batrachochytrium dendrobatidis* activity in bacterial isolates from three lowland species of atelopus. *PLoS ONE* 7 e44832

Fong JJ, Cheng TL, Bataille A, Pessier AP, Waldman B, Vredenburg VT (2015) Early 1900s detection of *Batrachochytrium dendrobatidis* in Korean amphibians. *PLoS ONE* 10 e0115656

Forrest MJ, Edwards MS, Rivera R, Sjöberg JC, Jaeger JR (2015) High prevalence and seasonal persistence of amphibian chytrid fungus infections in the desert-dwelling amargosa toad *Anaxyrus nelsoni*. *Herpetological Conservation and Biology* 10:917-925

Forrest MJ, Schlaepfer MA (2011) Nothing a hot bath won't cure: Infection rates of amphibian chytrid fungus correlate negatively with water temperature under natural field settings. *PLoS ONE* 6 e28444

Forti LR, Becker CG, Tacioli L, Pereira VR, Santos ACFA, Oliveira I, Haddad CFB, Toledo LF (2017) Perspectives on invasive amphibians in Brazil. *PLOS ONE* 12 e0184703

Forzan MJ, Vanderstiche R, Hogan NS, Teather K, Wood J (2010) Prevalence of *Batrachochytrium dendrobatidis* in three species of wild frogs on Prince Edward Island Canada. *Diseases of Aquatic Organisms* 91:91-96

Fox SF, Greer AL, Torres-Cervantes R, Collins JP (2006) First case of *Ranavirus*-associated morbidity and mortality in natural populations of the South American frog *Atelognathus patagonicus*. *Diseases of Aquatic Organisms* 72:87-92

Flechas SV, Vredenburg VT, Amézquita A (2015) Infection prevalence in three lowland species of harlequin toads from the threatened genus atelopus. *Herpetological Review* 46:528-532

Flechas SV, Paz A, Crawford AJ, Sarmiento C, Acevedo AA, Arboleda A, Bolívar-García W, Echeverry-Sandoval CL, Franco R, Mojica C, Muñoz A, Palacios-Rodríguez P, Posso-Terranova AM, Quintero-Marín P, Rueda-Solano LA, Castro-Herrera F, Amézquita A (2017) Current and predicted distribution of the pathogenic fungus *Batrachochytrium dendrobatidis* in Colombia a hotspot of amphibian biodiversity. *Biotropica* 49:685-694

Frias-Alvarez P, Vredenburg VT, Familiar-Lopez M, Longcore JE, Gonzalez-Bernal E, Santos-Barrera G, Zambrano L, Parra-Olea G (2008) Chytridiomycosis survey in wild and captive Mexican amphibians. *EcoHealth* 5:18-26

Freitas ES, Attapol R, Ampai N, Puanprapai P, Yodthong S, Termprayoon K, Siler CD, Aowphol A (2019) Amphibian surveys reveal no instances of *Batrachochytrium dendrobatidis* and suggest low prevalence of chytrid fungus. *Herpetological Review* 50:290-298

Gabor CR, Fisher MC, Bosch J (2013) A non-invasive stress assay shows that tadpole populations infected with *Batrachochytrium dendrobatidis* have elevated corticosterone levels. *PLoS ONE* 8 e56054

Gabriela Agostini M, Burrowes PA (2015) Infection patterns of the chytrid fungus, *Batrachochytrium dendrobatidis* on anuran assemblages in agro-ecosystems from Buenos Aires province Argentina. *Phyllomedusa* 14:113-126

Gabriela Agostini M, Cortelezzi A, Berkunsky I, Soler G, Burrowes P (2015) First record of *Batrachochytrium dendrobatidis* infecting threatened populations of tandilean red-belly toad (*Melanophryniscus aff. montevidensis*) in Argentina. *Revista Mexicana de Biodiversidad* 86:826-828

Gaertner JP, Forstner MRJ, O'Donnell L, Hahn D (2009) Detection of *Batrachochytrium dendrobatidis* in endemic salamander species from Central Texas. *EcoHealth* 6:20-26

Garcia MJ, Rodríguez-Brenes S, Kobisk A, Adler L, Ryan MJ, Taylor RC, Hunter KL (2019) Epigenomic changes in the túngara frog (*Physalaemus pustulosus*): Possible effects of introduced fungal pathogen and urbanization. *Evolutionary Ecology* 33:671-686

Garner TWJ, Walker S, Bosch J, Leech S, Rowcliffe JM, Cunningham AA, Fisher MC (2009) Life history tradeoffs influence mortality associated with the amphibian pathogen *Batrachochytrium dendrobatidis*. *Oikos* 118:783-791

Gaertner JP, McHenry D, Forstner MRJ, Hahn D (2010) Annual variation of *Batrachochytrium dendrobatidis* in the houston toad (*Bufo houstenensis*) and a sympatric congener (*Bufo nebulifer*). *Herpetological Review* 41:456-459

Gaertner JP, Mendoza JA, II, Neang T, Forstner MRJ, Hahn D (2011) Detection of *Batrachochytrium dendrobatidis* in frogs from different locations in Cambodia. *Herpetological Review* 42:546-549

Gaertner JP, Brown DJ, Mendoza JA, Forstner MRJ, Hahn D (2012) Geographic variation in *Batrachochytrium dendrobatidis* occurrence among populations of *Acris crepitans blanchardi* in Texas, USA. *Herpetological Review* 43:274-278

Garcia-Roa R, Sunyer J, Fernandez-Loras A, Bosch J (2014) First record of *Batrachochytrium dendrobatidis* in Nicaragua. *Herpetological Journal* 24:65-68

Gaulke CA, Irwin JT, Wagner RS (2011) Prevalence and distribution of *Batrachochytrium dendrobatidis* at montane sites in Central Washington state, USA. *Herpetological Review* 42:209-211

Ghirardi R, Lescano JN, Longo MS, Robledo G, Steciow MM, Perotti MG (2009) *Batrachochytrium dendrobatidis* in Argentina: First record in *Leptodactylus gracilis* and another record in Leptodactylus Ocellatus. *Herpetological Review* 40:175-176

Ghirardi R, Levy MG, Lopez JA, Corbalan V, Steciow MM, Gabriela Perotti M (2014) Endangered amphibians infected with the chytrid fungus *Batrachochytrium dendrobatidis* in austral temperate wetlands from Argentina. *Herpetological Journal* 24:129-133

Ghirardi R, López JA, Sanabria EA, Quiroga LB, Levy MG (2017) Pathogenic fungus in feral populations of the invasive North american bullfrog in Argentina. *Belgian Journal of Zoology* 147:81-86

Ghirardi R, López JA, Sanabria EA, Quiroga LB, Levy MG (2017) First report of *Batrachochytrium dendrobatidis* in the warty toad, *Rhinella spinulosa* (Wiegmann, 1834) from the Argentinean andean foothills. *Herpetological Review* 48:64–66

Ghirardi R, López JA, Antoniazzi CE (2018) The chytrid fungus *Batrachochytrium dendrobatidis* infecting the creole frog, *leptodactylus latrans*, in a new region of west Argentina. *Herpetological Review* 49:255–257

Glorioso BM, Waddle JH, Richards-Zawacki CL (2017) Prevalence of *Batrachochytrium dendrobatidis* and *B. salamandrivorans* in the gulf coast waterdog, *Necturus beyeri*, from Southeast Louisiana, USA. *Herpetological Review* 48:360–363

Gluesenkamp AG, Muscher-hodges BJ, Lee MM, Sandoval NM, Fenolio DB (2018) Sampling for *Batrachochytrium dendrobatidis* and *B. salamandrivorans* in the Texas Blind Salamander (*Eurycea rathbuni*). *Herpetological Review* 49:44–46

Goka K, Yokoyama J, Une Y, Kuroki T, Suzuki K, Nakahara M, Kobayashi A, Inaba S, Mizutani T, Hyatt AD (2009) Amphibian chytridiomycosis in Japan: Distribution, haplotypes and possible route of entry into Japan. *Molecular Ecology* 18:4757-4774

Goodman RM, Ararso YT (2012) Survey of *Ranavirus* and the fungus *Batrachochytrium dendrobatidis* in frogs of Central Virginia, USA. *Herpetological Review* 43:78-80

Goodman RM, Tyler JA, Reinartz DM, Wright AN (2019) Survey of *Ranavirus* and *Batrachochytrium dendrobatidis* in introduced frogs in Hawaii USA. *Journal of Wildlife Diseases* 55:668-672

Gonynor JL, Yabsley MJ, Jensen JB, 2011:a Preliminary survey of *Batrachochytrium dendrobatidis* exposure in hellbenders from a stream in Georgia, USA. *Herpetological Review* 42:58- 59

Goldberg TL, Readel AM, Lee MH (2007) Chytrid fungus in frogs from an equatorial African montane forest in Western Uganda. *Journal of Wildlife Diseases* 43:521-524

Goldberg CS, Hawley TJ, Waits LP (2009) Local and regional patterns of amphibian chytrid prevalence on the Osa Peninsula, Costa Rica. Herpetological Review 40:309-311

Gower DJ, Doherty-Bone T, Loader SP, Wilkinson M, Kouete MT, Tapley B, Orton F, Daniel OZ, Wynne F, Flach E, Mueller H, Menegon M, Stephen I, Browne RK, Fisher MC, Cunningham AA, Garner TWJ (2013) *Batrachochytrium dendrobatidis* Infection and lethal chytridiomycosis in caecilian amphibians (Gymnophiona). *EcoHealth* 10:173-183

Gower DJ, Doherty-Bone TM, Aberra RK, Mengistu A, Schwaller S, Menegon M, De Sa R, Saber SA, Cunningham AA, Loader SP (2012) High prevalence of the amphibian chytrid fungus (*Batrachochytrium dendrobatidis*) across multiple taxa and localities in the highlands of Ethiopia. *Herpetological Journal* 22:225-233

Gómez AV, Farr W, Hahn D, Dixon JR, Lazcano D, Forstner MRJ (2015) Absence of *Batrachochytrium dendrobatidis* in eighteen species of amphibians from a variety of habitats in Tamaulipas, mexico. *Herpetological Review* 46:34-37

Göçmen B, Veith M, Igci N, Akman B, Godmann O, Wagner N (2013) No detection of the amphibian pathogen *Batrachochytrium dendrobatidis* in terrestrial turkish salamanders (Lyciasalamandra) despite its occurrence in syntopic frogs (*Pelophylax bedriagae*). *Salamandra* 49:51-55

Graham CM, Narayan EJ, McCallum H, Hero J-M (2013) Non-invasive monitoring of glucocorticoid physiology within highland and lowland populations of native Australian great barred frog (*Mixophyes fasciolatus*). *General and Comparative Endocrinology* 191:24-30

Gratwicke B, Alonso A, Elie T, Kolowski J, Lock J, Rotzel N, Sevin J, Fleischer RC (2011a) *Batrachochytrium dendrobatidis* not detected on amphibians from two lowland sites in Gabon Africa. *Herpetological Review* 42:69-71

Gratwicke B, Evans M, Grant EHC, Greathouse J, McShea WJ, Rotzel N, Fleischer RC (2011b) Low prevalence of *Batrachochytrium dendrobatidis* detected in appalachian salamanders from Warren County, Virginia, USA. *Herpetological Review* 42:217-219

Green D, Sherman C (2001) Diagnostic Histological findings in yosemite toads (*Bufo Canorus*) from a die-off in the 1970s. *Journal of Herpetology* 35:92-103

Greenbaum E, Kusamba C, Aristote MM, Reed K (2008) Amphibian chytrid fungus infections in hyperolius (Anura: Hyperoliidae) from Eastern Democratic Republic of Congo. *Herpetological Review* 39:70-73

Greenbaum E, Meece J, Reed KD, Kusamba C (2014) Amphibian chytrid infections in non-forested Habitats of Katanga, Democratic Republic of the Congo. *Herpetological Review* 45: 610-614

Greenbaum E, Meece J, Reed KD, Kusamba C (2015) Extensive occurrence of the amphibian chytrid fungus in the albertine rift a Central African amphibian hotspot. *Herpetological Journal* 25:91-100

Greener MS, Shepherd R, Hoskisson PA, Asmath H, Downie JR (2017) How many trinidad stream frogs (*Mannophryne trinitatis*) are there, and should they be regarded as vulnerable to extinction? *Herpetological Journal* 27:5-11

Greenhawk N, Zlotnik S, Billy LM, Boas S, Gabel S (2017) Baseline amphibian survey and sampling of *Batrachochytrium dendrobatidis* in the Icaco and Hormiga Valleys Patillas Puerto Rico Phyllomedusa: J*ournal of Herpetology* 16:63-69

Greenspan SE, Roznik EA, Schwarzkopf L, Alford RA, Pike DA (2016) Robust calling performance in frogs infected by a deadly fungal pathogen. *Ecology and Evolution* 6:5964-5972

Grogan LF, Phillott AD, Scheele BC, Berger L, Cashins SD, Bell SC, Puschendorf R, Skerratt LF (2016) Endemicity of chytridiomycosis features pathogen overdispersion. *Journal of Animal Ecology* 85:806-816

Grogan LF, Cashins SD, Skerratt LF, Berger L, McFadden MS, Harlow P, Hunter DA, Scheele BC, Mulvenna J (2018) Evolution of resistance to chytridiomycosis is associated with a robust early immune response. *Molecular Ecology* 27:919-934

Groner ML, Relyea RA (2010) *Batrachochytrium dendrobatidis* is present in Northwest Pennsylvania, USA, with high prevalence in *Notophthalums viridescens*. *Herpetological Review* 41:462-465

Gruendler MC, Toledo LF, Parra-Olea G, Haddad CFB, Giasson LOM, Sawaya RJ, Prado CPA, Araujo OGS, Zara FJ, Centeno FC, Zamudio KR (2012) Interaction between breeding habitat and elevation affects prevalence but not Infection intensity of *Batrachochytrium dendrobatidis* in Brazilian anuran assemblages. *Diseases of Aquatic Organisms* 97:173-184

Grummer JA, Leache AD (2016) Survey for *Batrachochytrium dendrobatidis* in the North Cascades National Park Service Complex Washington, USA. *Herpetological Review* 47:392-394

Guayasamin JM, Mendoza AM, Longo AV, Zamudio KR, Bonaccorso E (2014) High prevalence of *Batrachochytrium dendrobatidis* in an andean Frog Community (Reserva Las Gralarias, Ecuador). *Amphibian, Reptile Conservation* 8:33-44

Guthrie A, Sweeney R, Steele K (2017) *Batrachochytrium dendrobatidis* and *Batrachochytrium salamandrivorans* surveillance in salamanders of Southeastern Virginia, USA. *Herpetological Review* 48:363–365

Gutierrez FR, Arellano ML, Moreno LE, Natale GS (2010) *Batrachochytrium dendrobatidis* in Argentina: First record of infection in *Hypsiboas cordobae* and *Odontophrynus occidentalis* tadpoles in San Luis Province. *Herpetological Review* 41:323-325

Gutsche A, McCranie JR, Ohst T, Orellana LV (2015) New Records of the Chytrid Fungus *Batrachochytrium dendrobatidis* in Honduran Frogs. *Herpetological Review* 46:202-205

Hammond TT, Blackwood PE, Shablin SA, Richards-Zawacki CL (2020) Relationships between glucocorticoids and infection with *Batrachochytrium dendrobatidis* in three amphibian species. *General and Comparative Endocrinology* 285 113269

Hanlon SM, Smith D, Kerby JL, Berg E, Peterson W, Parris MJ, Moore JE (2014a) Occurrence of *Batrachochytrium dendrobatidis* in Wapanocca National Wildlife Refuge Arkansas, USA. *Herpetological Review* 45:31-32

Hanlon SM, Smith D, Kerby JL, Parris MJ, Moore JE (2014b) Detection of *Batrachochytrium dendrobatidis* infections in amphibian populations at Edward J. Meeman biological field Station Tennessee, USA. *Herpetological Review* 45:32-34

Hanselmann R, Rodriguez A, Lampo M, Fajardo-Ramos L, Aguirre A, Kilpatrick A, Rodriguez J, Daszak P (2004) Presence of an emerging pathogen of amphibians in introduced bullfrogs *Rana catesbeiana* in Venezuela. *Biological Conservation* 120:115-119

Harner MJ, Nelson AJ, Geluso K, Simon DM (2011) Chytrid Fungus in American bullfrogs (*Lithobates catesbeianus*) along the Platte River Nebraska, USA. *Herpetological Review* 32: 549-551

Heard GW, Scroggie MP, Clemann N, Ramsey DSL (2014) Wetland characteristics influence disease risk for a threatened amphibian. *Ecological Applications* 24:650-662

Herrera R, Steciow M, Natale G (2005) Chytrid fungus parasitizing the wild amphibian *Leptodactylus ocellatus* (Anura : Leptodactylidae) in Argentina. *Diseases of Aquatic Organisms* 64:247-252

Hernández-Gómez O, Byrne AQ, Gunderson AR, Jenkinson TS, Noss CF, Rothstein AP, Womack MC, Rosenblum EB (2020) Invasive vegetation affects amphibian skin microbiota and body condition. *PeerJ* 8 e8549

Hernández-Martínez LÁ, Romero-Méndez U, González-Barrios JL, García-De la Peña MC, Amézquita-Torres A (2019) Nuevos registros y prevalencia de *Batrachochytrium dendrobatidis* en anuros d la Cuenca Nazas-Aguanaval en la región norte-centro de México. *Revista Mexicana de Biodiversidad* 90 e902934

Hidalgo-Vila J, Diaz-Paniagua C, Marchand MA, Cunningham AA (2012) *Batrachochytrium dendrobatidis* infection of amphibians in the Donana National Park, Spain. *Diseases of Aquatic Organisms* 98:113-119

Hill RL, Levy MG, Timpe EK, Kaylock JB (2011) Additional reports of the amphibian chytrid fungus *Batrachochytrium dendrobatidis* from Georgia, USA. *Herpetological Review* 42:376-378

Hill RL, Levy MG (2014) Prevalence of *Batrachochytrium dendrobatidis* in pond-breeding amphibians of the Fall Line Sandhills region of Georgia, USA. *Herpetological Review* 45:236-238

Hirschfeld M, Blackburn DC, Doherty-Bone TM, Gonwouo LN, Ghose S, Rödel M-O (2016) Dramatic declines of montane frogs in a Central African biodiversity hotspot. *PLoS ONE* 11 e0155129

Hite JL, Bosch J, Fernandez-Beaskoetxea S, Medina D, Hall SR (2016) Joint effects of habitat zooplankton host stage structure and diversity on amphibian chytrid. *Proceedings of the Royal Society B: Biological Sciences* 283:20160832

Holmes I, McLaren K, Wilson B (2012) Surveys for frog diversity and *Batrachochytrium dendrobatidis* in Jamaica. *Herpetological Review* 43:278-282

Holmes I, McLaren K, Wilson B (2014) Precipitation constrains amphibian chytrid fungus infection rates in a terrestrial frog assemblage in Jamaica West Indies. *Biotropica* 46:219-228

Hopkins S, Channing A (2003) Chytrid Fungus in Northern and Western Cape Frog Populations South Africa. *Herpetological Review* 34:334-336

Horner AA, Hoffman EA, Tye MR, Hether TD, Savage AE (2017) Cryptic chytridiomycosis linked to climate and genetic variation in amphibian populations of the Southeastern United States. *PLOS ONE* 12 e0175843

Hoskin CJ, Hines HB, Webb RJ, Skerratt LF, Berger L (2018) Naïve rainforest frogs on Cape York Australia are at risk of the introduction of amphibian chytridiomycosis disease. *Australian Journal of Zoology* 66:174-178

Hossack BR, Adams MJ, Grant EHC, Pearl CA, Bettaso JB, Barichivich WJ, Lowe WH, True K, Ware JL, Corn PS (2010) Low prevalence of chytrid fungus (*Batrachochytrium dendrobatidis*) in amphibians of us headwater streams. *Journal of Herpetology* 44:253-260.

Hossack BR, Lowe WH, Ware JL, Corn PS (2013) Disease in a dynamic landscape: Host behavior and wildfire reduce amphibian chytrid infection. *Biological Conservation* 157:293-299

Hoverman JT, Mihaljevic JR, Richgels KLD, Kerby JL, Johnson PTJ (2012) Widespread co-occurrence of virulent pathogens within California amphibian communities. *EcoHealth* 9:288-292

Huang R, Wilson LA (2013) *Batrachochytrium dendrobatidis* in amphibians of the piedmont and blue ridge provinces in Northern Georgia, USA. *Herpetological Review* 44:95-98

Hudson MA, Young RP, Jackson JD, Orozco-terWengel P, Martin L, James A, Sulton M, Garcia G, Griffiths RA, Thomas R, Magin C, Bruford MW, Cunningham AA (2016) Dynamics and genetics of a disease-driven species decline to near extinction: Lessons for conservation. *Scientific Reports* 6:40110

Hudson MA, Griffiths RA, Martin L, Fenton C, Adams S.-L, Blackman A, Sulton M, Perkins MW, Lopez J, Garcia G, Tapley B, Young RP, Cunningham AA (2019) Reservoir frogs: Seasonality of *Batrachochytrium dendrobatidis* infection in robber frogs in Dominica and Montserrat. *PeerJ* 7 e7021

Hughey MC, Becker MH, Walke JB, Swartwout MC, Belden LK (2014) *Batrachochytrium dendrobatidis* in Virginia amphibians: Within and among site variation in infection. *Herpetological Review* 45:428-438

Hughey MC, Sokol ER, Walke JB, Becker MH, Belden LK (2019) Ecological correlates of large-scale turnover in the dominant members of *Pseudacris crucifer* skin bacterial communities. *Microbial Ecology* 78:832-842

Huss M, Huntley L, Vredenburg V, Johns J, Green S (2013) Prevalence of *Batrachochytrium dendrobatidis* in 120 archived specimens of *Lithobates catesbeianus* (American bullfrog) collected in California 1924-2007. *EcoHealth* 10:339-343

Hydeman ME, Bell RC, Drewes RC, Zamudio KR (2013) Amphibian chytrid fungus confirmed in endemic frogs and caecilians on the island of Sãotomé, Africa. *Herpetological Review* 44:254-257

Hyman OJ, Collins JP (2015) *Batrachochytrium dendrobatidis* dynamics in an isolated Northern leopard frog (*Lithobates pipiens*) population in Arizona. *Herpetological Review* 46:535-537

Hyne RV, Spolyarich N, Wilson SP, Patra RW, Byrne M, Gordon G, Sanchez-Bayo F, Palmer CG (2009) Distribution of frogs in rice bays within an irrigated agricultural area: Links to pesticide usage and farm practices. *Environmental Toxicology and Chemistry* 28:1255-1265

Igleski MJ, Nicholson KE (2014) Spatial pattern of *Batrachochytrium dendrobatidis* infection in green frogs (Lithobates clamitans) in Michigan, USA. *Herpetological Review* 45:34-40

Imasuen A, Aisien MSO, Weldon C, Dalton D, Kotzé, A, De Preez LH (2011) Occurrence of *Batrachochytrium dendrobatidis* in amphibian populations of Okomu national park Nigeria. *Herpetological Review* 42:379-382

Jacinto-Maldonado M, García-Peña GE, Paredes-León R, Saucedo B, Sarmiento-Silva RE, García A, Martínez-Gómez D, Ojeda M, Del Callejo E, Suzán G (2020) Chiggers (Acariformes: Trombiculoidea) do not increase rates of infection by *Batrachochytrium dendrobatidis* fungus in the endemic dwarf Mexican treefrog *Tlalocohyla smithii* (Anura: Hylidae). *International Journal for Parasitology: Parasites and Wildlife* 11:163-173

Jaeger JR, Waddle AW, Rivera R, Harrison DT, Ellison S, Forrest MJ, Vredenburg VT, van Breukelen F (2017) *Batrachochytrium dendrobatidis* and the decline and survival of the relict leopard frog. *Ecohealth* 14:285-295

Jani AJ, Knapp RA, Briggs CJ (2017) Epidemic and Endemic Pathogen Dynamics Correspond to Distinct Host Population Microbiomes at a Landscape Scale. *Proceedings of the Royal Society B: Biological Sciences* 284 20170944

Jenkinson TS, Roman CMB, Lambertini C, Valencia-Aguilar A, Rodriguez D, Nunes-de-Almeida CHL, Ruggeri J, Belasen AM, Da Silva Leite D, Zamudio KR, Longcore JE, Toledo LF, James TY (2016) Amphibian-killing chytrid in Brazil comprises both locally endemic and globally expanding populations. *Molecular Ecology* 25:2978-2996

Jiménez RR, Alvarado G, Estrella J, Sommer S (2019) Moving beyond the host: Unraveling the skin microbiome of endangered costa rican amphibians. *Frontiers in Microbiology* 10 2060

Johnson PTJ, McKenzie VJ, Peterson AC, Kerby JL, Brown J, Blaustein AR, Jackson T (2011) Regional decline of an iconic amphibian associated with elevation land-use change and invasive species. *Conservation Biology* 25:556-566

Johnson PTJ, Calhoun DM, Stokes AN, Susbilla CB, McDevitt-Galles T, Briggs CJ, Hoverman JT, Tkach VV, de Roode JC (2018) Of Poisons and Parasites-the Defensive Role of Tetrodotoxin Against Infections in Newts. *Journal of Animal Ecology* 87:1192-1204

Jongsma GFM, Kaya AB, Yoga J-A, Mbega J-D, Beh J-HM, Tobi E, Emrich AM, Dixon-MacCallum GP, Davis DR, Kerby JL, Blackburn DC (2016) Widespread presence and high prevalence of *Batrachochytrium dendrobatidis* in Gabon. *Herpetological Review* 47:227-230

Jongsma GFM, Empey MA, Smme CM, Bennett AM, McAlpine DE (2019) High prevalence of the amphibian pathogen *Batrachochytrium dendrobatidis* in plethodontid salamanders in protected areas in New Brunswick, Canada. 14:91-96

Joseph MB, Knapp RA (2018) Disease and climate effects on individuals drive post-reintroduction population dynamics of an endangered amphibian. *Ecosphere* 9 e02499

Julian J, Gould V, Glenney G, Brooks R (2016) Seasonal infection rates of *Batrachochytrium dendrobatidis* in populations of Northern green frog *Lithobates clamitans melanota* tadpoles. *Diseases of Aquatic Organisms* 121:97-104

Kadekaru S, Tamukai K, Tominaga A, Goka K, Une Y (2016) Spontaneous oral chytridiomycosis in wild bullfrog tadpoles in Japan. *Journal of Veterinary Medical Science* 78:573-577

Kaiser K, Grafe UT. (2011) Chytrid fungus not found in preliminary survey of lowland amphibian populations across Northwestern Borneo. *Herpetological Review* 42:59-61

Kaiser K, Pollinger J (2012) *Batrachochytrium dendrobatidis* shows high genetic diversity and ecological niche specificity among haplotypes in the Maya Mountains of Belize. *PLoS ONE* 7 e32113

Karvemo S, Meurling S, Berger D, Höglund J, Laurila A (2018) Effects of host species and environmental factors on the prevalence of *Batrachochytrium dendrobatidis* in Northern Europe. *PLOS ONE* 13 e0199852

Karvemo S, Wikstrom G, Widenfalk LA, Hoglund J, Laurila A (2020) Chytrid fungus dynamics and infections associated with movement distances in a red-listed amphibian. *Journal of Zoology doi:10.1111/jzo.12773*

Kielgast J, Rödder D, Veith M, Lötters S (2010) Widespread occurrence of the amphibian chytrid fungus in Kenya. *Animal Conservation* 13:36-43

Kiemnec-Tyburczy KM, Eddy SL, Chuinard AJ, Houck LD (2012) Low prevalence of *Batrachochytrium dendrobatidis* in two plethodontid salamanders from North Carolina, USA. *Herpetological Review* 43:85-87

Kindermann C, Narayan EJ, Hero J-M (2012) Urinary corticosterone metabolites and chytridiomycosis disease prevalence in a free-living population of male stony creek frogs (*Litoria wilcoxii*). *Comparative Biochemistry and Physiology Part A: Molecular, Integrative Physiology* 162:171-176

Kindermann C, Narayan EJ, Hero JM (2017) Does physiological response to disease incur cost to reproductive ecology in a sexually dichromatic amphibian species? *Comparative Biochemistry and Physiology Part A: Molecular, Integrative Physiology* 203 220-226

Kinney VC, Heemeyer JL, Pessier AP, Lannoo MJ (2011) Seasonal pattern of *Batrachochytrium dendrobatidis* infection and mortality in *Lithobates areolatus*: Affirmation of Vredenburg's "10,000 Zoospore Rule." *PLoS ONE* 6 e16708

Klemish JL, Johnson BL, Siddons SR, Wild ER (2012) Occurrence of *Batrachochytrium dendrobatidis* among populations of *Lithobates clamitans* and *L. pipiens* in Wisconsin, USA. *Herpetological Review* 43:282-288

Knapp RA, Briggs CJ, Smith TC, Maurer JR (2011) Nowhere to hide: Impact of a temperature-sensitive amphibian pathogen along an elevation gradient in the temperate zone. *Ecosphere* 2:UNSP 93

Köhler G, Than NL, Flomm A, Eisenberg T (2019) A preliminary survey for *Batrachochytrium dendrobatidis* in Myanmar. *Herpetological Review* 50:298-300

Köhler G, Georg Hantke, Méndez-de La Cruz FR, Flamm A, Eisenberg T (2019) A survey for the amphibian chytrid fungus *Batrachochytrium dendrobatidis* in the Mexican states of Mexico, Morelos, Oaxaca, and Puebla. *Herpetological Review* 50:303-305

Kolby JE, Padgett-Flohr GE, Field R (2010) Amphibian chytrid fungus *Batrachochytrium dendrobatidis* in cusuco national park Honduras. *Diseases of Aquatic Organisms* 92:245-251.

Kosch TA, Morales V, Summers K (2012) *Batrachochytrium dendrobatidis* in Peru. *Herpetological Review* 43:288-293

Korfel CA, Hetherington TE (2014) Temperature alone does not explain patterns of *Batrachochytrium dendrobatidis* infections in the green frog *lithobates clamitans*. *Diseases of Aquatic Organisms* 109:177-185

Kriger KM, Hero J-M (2008) Altitudinal distribution of chytrid (*Batrachochytrium dendrobatidis*) infection in subtropical Australian frogs. *Austral Ecology* 33:1022-1032

Kriger Kerry M, Hero J-M (2007) The chytrid fungus *Batrachochytrium dendrobatidis* is non-randomly distributed across amphibian breeding habitats. *Diversity and Distributions* 13:781-788

Kriger K. M, Hero J-M (2007) Large-scale seasonal variation in the prevalence and severity of chytridiomycosis. *Journal of Zoology* 271:352-359

Kriger KM, Hero J-M (2006) Survivorship in wild frogs infected with chytridiomycosis. *EcoHealth* 3:171-177

Kriger KM, Hero J-M, Ashton KJ (2006a) Cost efficiency in the detection of chytridiomycosis using PCR assay. *Diseases of Aquatic Organisms* 71:149-154

Kriger KM, Hines HB, Hyatt AD, Boyle DG, Hero J-M (2006b) Techniques for detecting chytridiomycosis in wild frogs: Comparing histology with real-time Taqman PCR. *Diseases of Aquatic Organisms* 71:141-148

Kriger KM, Pereoglou F, Hero J-M (2007) Latitudinal variation in the prevalence and intensity of chytrid (*Batrachochytrium dendrobatidis*) infection in Eastern Australia. *Conservation Biology* 21:1280-1290

Krynak TJ, Robinson TL, Scott JJ (2012) Detection of *Batrachochytrium dendrobatidis* in amphibian populations of Northeast Ohio. *Herpetological Review* 43:87-89

Krynak KL, Wessels DG, Snyder EB, Krynak TJ, Imba S, Lyons JA, Loudon AH, Guayasamin JM (2018) A preliminary assessment of the skin-associated microbiome of *Caecilia buckleyi* (Amphibia: Caeciliidae) 49(4) 690–693

Kueneman JG, Weiss S, McKenzie VJ (2017) Composition of micro-eukaryotes on the skin of the cascades frog (*Rana cascadae*) and patterns of correlation between skin microbes and *Batrachochytrium dendrobatidis. Frontiers in Microbiology* 8 2350

Kusrini MD, Skerratt LF, Garland S, Berger L, Endarwin W (2008) Chytridiomycosis in frogs of Mount Gede Pangrango, Indonesia. *Diseases of Aquatic Organisms* 82:187-194

Labisko J, Maddock ST, Taylor M, Chong-Seng L, Gower D, Wynne FJ, Wormwell E, Morel C, French GCA, Bunbury N, Bradfield KS (2015) Chytrid fungus (*Batrachochytrium dendrobatidis*) undetected in the two orders of Seychelles amphibians. *Herpetological Review* 45:41-45

Laking AE, Ngo HN, Pasmans F, Martel A, Nguyen TT (2017) *Batrachochytrium salamandrivorans* is the predominant chytrid fungus in vietnamese salamanders. *Scientific Reports* 7 44443

Lambert BA, Schorr RA, Schneider SC, Muths E (2016) Influence of demography and environment on persistence in toad populations. *Journal of Wildlife Management* 80:1256-1266

Lambertini C, Guilherme Becker C, Jenkinson TS, Rodriguez D, Leite D. Da S, James TY, Zamudio KR, Toledo LF (2016) Local phenotypic variation in amphibian-killing fungus predicts infection dynamics. *Fungal Ecology* 20:15-21

Lampo M, Barrio-Amoros C, Han B (2006a) *Batrachochytrium dendrobatidis* infection in the recently rediscovered *Atelopus mucubajiensis* (Anura bufonidae) a critically endangered frog from the venezuelan Andes. *EcoHealth* 3:299-302

Lampo M, Rodriguez-Contreras A, La Marca E, Daszak P (2006b) A chytridiomycosis epidemic and a severe dry season precede the disappearance of atelopus species from the venezuelan Andes. *Herpetological Journal* 16:395-402

Lampo M, Seraris CJ (2006c) Unexplained amphibian mortalities in the secluded mountains of the venezuelan Guayana: Is there evidence of chytridiomycosis? *Herpetological Review* 37:47-49

Lampo M, Sánchez D, Nicolás A, Márquez M, Nava-González F, García CZ, Rinaldi M, Rodríguez-Contreras A, León F, Han BA, Chacón-Ortiz A (2008) *Batrachochytrium dendrobatidis* in Venezuela. *Herpetological Review* 39:449-454

Lampo M, Señaris C, García CZ (2017) Population Dynamics of the critically endangered toad *Atelopus cruciger* and the fungal disease chytridiomycosis. *PLOS ONE* 12 e0179007

Lane E, Weldon C, Bingham J (2003) Histological evidence of chytridiomycete fungal infection in a free-ranging amphibian *Afrana fuscigula* (Anura : Ranidae) in South Africa. *Journal of the South African Veterinary Association* 74:20-21

Langhammer PF, Burrowes PA, Lips KR, Bryant AB, Collins JP (2014) Susceptibility to the amphibian chytrid fungus varies with ontogeny in the direct-developing frog *Eleutherodactylus coqui Journal of Wildlife Diseases* 50:438-446

Lannoo MJ, Petersen C, Lovich RE, Nanjappa P, Phillips C, Mitchell JC, Macallister I (2011) Susceptibility to the amphibian chytrid fungus varies with ontogeny in the direct-developing frog US transect reveals spatial and temporal patterns of *Batrachochytrium dendrobatidis* infection. *PLoS ONE* 6 e22211

Lawson TD, Jones ML, Komar O, Welch AM (2011) Prevalence of *Batrachochytrium dendrobatidis* in *Agalchnis moreetii* (Hylidae) of El Salvador and association with larval jaw sheath depigmentation. *Journal of Wildlife Diseases* 47:544-554

LeBlanc J, Faruk A, Dort E, Govindarajulu P, Quah E, Muin MA, Hintz W (2014) Multi-year surveillance for *Batrachochytrium dendrobatidis* in amphibians of peninsular Malaysia. *Herpetological Review* 45:603-608

Lenker MA, Savage AE, Becker CG, Rodriguez D, Zamudio KR (2014) *Batrachochytrium dendrobatidis* infection dynamics vary seasonally in upstate New York, USA. *Diseases of Aquatic Organisms* 111:51-60

Lennon C, Hudman SP, Montgomery CE (2014) Assessment of *Batrachochytrium dendrobatidis* Infection Level in Amphibians of Wakonda State Park Missouri, USA. *Herpetological Review* 45:40

Lescano JN, Longo S, Robledo G (2013) Chytridiomycosis in endemic amphibians of the mountaintops of the Cordoba and San Luis Ranges Argentina. *Diseases of Aquatic Organisms* 102:249-254

Lindquist ED, Shin MJ, Cossel Jr JO, Suckert AMM, Bletz MC, Trimmer NC (2011) Chytrid in a canopy amphibian: Picado's bromeliad treefrog *Isthmohyla picadoi* (Hylidae) persists at a site affected by *Batrachochytrium dendrobatidis*. *Herpetological Review* 42:205-208

Lindquist E, Cossel J, Jr Cordoba KL, Salamanca J, Zambana I, McFarland HR, Luthman K (2016) First record of the pathogenic fungus *Batrachochytrium dendrobatidis* in *Hypsiboas riojanus* and H. callipleura from Central Bolivia (Anura: Hylidae). *Herpetological Review* 47:400-402

Lips K, Green D, Papendick R (2003) Chytridiomycosis in wild frogs from Southern Costa Rica. *Journal of Herpetology* 37:215-218

Lips K, Mendelson J, Munoz-Alonso A, Canseco-Marquez L, Mulcahy D (2004) Amphibian population declines in montane Southern Mexico: Resurveys of historical localities. *Biological Conservation* 119:555-564

Lips K, Brem F, Brenes R, Reeve J, Alford R, Voyles J, Carey C, Livo L, Pessier A, Collins J (2006) Emerging infectious disease and the loss of biodiversity in a neotropical amphibian community. *Proceedings of the National Academy of Sciences of the United States of America.* 103:3165-3170

Lisboa BS, De Moura Neves JM, Cavalcanti Do Nascimento FA, Tavares-Bastos L, Mott T (2013) New records of *Batrachochytrium dendrobatidis* in the Atlantic Forest of Northeastern Brazil. *North-Western Journal of Zoology* 9:210-213

Longcore JR, Longcore JE, Pessier AP, Halteman WA (2007) Chytridiomycosis Widespread in anurans of Northeastern United States. *Journal of Wildlife Management* 71:435-444

Longo AV, Burrowes PA (2010) Persistence with chytridiomycosis does not assure survival of direct-developing frogs. *EcoHealth* 7:185-195
Longo AV, Burrowes PA, Joglar RL (2010) Seasonality of *Batrachochytrium dendrobatidis* infection in direct-developing frogs suggests a mechanism for persistence. *Diseases of Aquatic Organisms* 92:253-260
Longo AV, Ossiboff RJ, Zamudio KR, Burrowes PA (2013) Lability in host defenses: Terrestrial frogs die from chytridiomycosis under enzootic conditions. *Journal of Wildlife Diseases* 49:197-199
Longo AV, Savage AE, Hewson I, Zamudio KR (2015) Seasonal and ontogenetic variation of skin microbial communities and relationships to natural disease dynamics in declining amphibians. *Royal Society Open Science* 2:140377
Longo AV, Zamudio KR (2017) Temperature variation bacterial diversity and fungal infection dynamics in the amphibian skin. *Molecular Ecology* 26:4787-4797
Longo AV, Fleischer RC, Lips KR (2019) Double trouble: Co-infections of chytrid fungi will severely impact widely distributed newts. *Biological Invasions* 21:2233-2245
Love CN, Winzeler ME, Beasley R, Scott DE, Nunziata SO, Lance SL (2016) Patterns of amphibian infection prevalence across wetlands on the Savannah River site South Carolina, USA. *Diseases of Aquatic Organisms* 121:1-14
Lovich RE, Akre T, Ryan M, Nuñez S, Cruz G, Borjas G, Scott NJ, Flores S, Del Cid W, Flores A, Rodriguez C, Luque-Montes IR, Ford R (2010) New herpetofaunal records from Southern Honduras. *Herpetological Review* 41:112-115
Luja VH, Rodriguez-Estrella R, Ratzlaff K, Parra-Olea G, Ramirez-Bautista A (2012) The chytrid fungus *Batrachochytrium dendrobatidis* in isolated populations of the Baja California treefrog Pseudacris *Hypochondriaca curta* in Baja California Sur, Mexico. South*western Naturalist* 57:323-327
Luría-Manzano R, Canseco-Márquez L, Frías-Alvarez P (2011) *Batrachochytrium dendrobatidis* in *Plectrohyla arborescandens* (Anura: Hylidae) larvae at a montane site in the Sierra Negra Puebla, Mexico. *Herpetological Review* 42:552-554
Mali I, Villamizar-Gomez A, Krizmanić I, Ajtić R, Forstner MRJ (2017) Evidence of *Batrachochytrium dendrobatidis* Infection in Amphibians from Serbian Lowlands. *Journal of Wildlife Diseases* 53:686-689
Martinez Rodriguez E, Gamble T, Hirt VM, Cotner S (2009) Presence of *Batrachochytrium dendrobatidis* at the headwaters of the Mississippi River, Itasca State Park, Minnesota, USA. *Herpetological Review* 40:48-50
Martel A, Adriaensen C, Sharifian-Fard M, Spitzen-van Der Sluijs A, Louette G, Baert K, Crombaghs B, Dewulf J, Pasmans F (2013) The absence of zoonotic agents in invasive bullfrogs (*Lithobates catesbeianus*) in Belgium and the Netherlands. *EcoHealth* 10:344-347
Martel A, Blooi M, Adriaensen C, Van Rooij P, Beukema W, Fisher MC, Farrer RA, Schmidt BR, Tobler U, Goka K, Lips KR, Muletz C, Zamudio KR, Bosch J, Lötters S, Wombwell E, Garner TWJ, Cunningham AA, Spitzen-van Der Sluijs A, Salvidio S, Ducatelle R, Nishikawa K, Nguyen TT, Kolby JE, Van Bocxlaer I, Bossuyt F, Pasmans F (2014) Recent introduction of a chytrid fungus endangers Western palearctic salamanders. *Science* 346:630-631
Martel A, Fard MS, Van Rooij P, Jooris R, Boone F, Haesebrouck F, Van Rooij D, Pasmansl F (2012) Road-killed common toads (*Bufo bufo*) in Flanders (Belgium) reveal low prevalence of *Ranavirus*es and *Batrachochytrium dendrobatidis*. *Journal of Wildlife Diseases* 48:835-839
McCracken S, Gaertner JP, Forstner MRJ, Hahn D (2009) Detection of *Batrachochytrium dendrobatidis* in amphibians from the forest floor to the upper canopy of an Ecuadorian Amazon lowland rainforest. *Herpetological Review* 40:190-194
McLeod DS, Sheridan JA, Jiraungkoorskul W, Khonsue W (2008) A Survey for chytrid fungus in Thai Amphibians. *Raffles Bulletin of Zoology* 56:199-204

McLelland DJ, Smith IP, Olds L, Myers C, Skerratt LF (2013) *Batrachochytrium dendrobatidis* not detected in anurans in the Northern Kimberley region of Western Australia. *Herpetological Review* 44:98-100

McMillan KM, Lesbarrères D, Harrison XA, Garner TWJ (2020) Spatiotemporal heterogeneity decouples infection parameters of amphibian chytridiomycosis. *Journal of Animal Ecology* 89:1109-1121

McTaggart AL, Eberl T, Keller O, Jameson ML (2014) First report of *Batrachochytrium dendrobatidis* associated with amphibians in Kansas, USA. *Herpetological Review* 45:439-441

Medina D, Garner TWJ, Carrascal LM, Bosch J (2015) Delayed metamorphosis of amphibian larvae facilitates *Batrachochytrium dendrobatidis* transmission and persistence. *Diseases of Aquatic Organisms* 117:85-92

Medina D, Hughey MC, Walke JB, Becker MH, Pontarelli K, Sun S, Badgley B, Belden LK (2019) Amphibian skin fungal communities vary across host species and do not correlate with infection by a pathogenic fungus. *Environmental Microbiology* 21:2905-2920

Mendelson JR, III, Jones MEB, Pessier AP, Toledo G, Kabay EH, Campbell JA (2014) On the timing of an epidemic of amphibian chytridiomycosis in the highlands of Guatemala. South *American Journal of Herpetology* 9:151-153

Mendoza JA, II, Gaertner JP, Holden J, Forstner MRJ, Hahn D (2011) Detection of *Batrachochytrium dendrobatidis* on amphibians in Pursat province Cambodia. *Herpetological Review* 42:542-545

Mendoza-Almeralla C, Lopez-Velazquez A, Longo AV, Parra-Olea G (2016) Temperature treatments boost subclinical infections of *Batrachochytrium dendrobatidis* in a Mexican salamander (*Pseudoeurycea leprosa*). *Revista Mexicana de Biodiversidad* 87:171-179

Miaud C, Pozet F, Gaudin NCG, Martel An, Pasmans F, Labrut S (2016) *Ranavirus* causes mass die-offs of alpine amphibians in the Southwestern Alps, France. Journal of Wildlife Diseases 52:242-252

Miller CA, Tasse Taboue GC, Ekane MMP, Robak M, Sesink Clee PR, Richards-Zawacki C, Fokam EB, Fuashi NA, Anthony NM (2018) Distribution Modeling and Lineage Diversity of the Chytrid Fungus *Batrachochytrium dendrobatidis* (*Bd*) in a Central African Amphibian Hotspot. *PLOS ONE* 13 e0199288

Moffitt D, Williams LA, Hastings A, Pugh MW, Gangloff MM, Siefferman L (2015) Low prevalence of the amphibian pathogen *Batrachochytrium dendrobatidis* in the Southern Appalachian Mountains. *Herpetological Conservation and Biology* 10:123-136

Molur S, Krutha K, Paingankar MS, Dahanukar N (2015) Asian strain of *Batrachochytrium dendrobatidis* is widespread in the Western Ghats, India. *Diseases of Aquatic Organisms* 112:251-255

Monsen-Collar K, Hazard L, Dussa R (2010) Comparison of PCR and rt-PCR in the first report of *Batrachochytrium dendrobatidis* in amphibians in New Jersey, USA. *Herpetological Review* 41:460-462

Moreno V, Aguayo CA, Brunton DH (2011) A Survey for the amphibian chytrid fungus *Batrachochytrium dendrobatidis* in New Zealand's endemic Hochstetter's Frog (*Leiopelma hochstetteri*). *New Zealand Journal Zoology* 38:181-184

Mowry CB, Keene CM, Prisland SE, Tyler BD, Montgomery AA, Mowry AP, Martin RA, Stevens S, Ellwanger J Morgan MB (2017) A survey of *Batrachochytrium dendrobatidis* occurrence in amphibians of Walker and Floyd Counties, Georgia, USA. *Herpetological Review* 48:777-779

Muelleman PJ, Montgomery CE (2013) *Batrachochytrium dendrobatidis* in amphibians of Northern Calhoun County, Illinois, USA. *Herpetological Review* 44:614-615

Muletz C, Caruso NM, Fleischer RC, McDiarmid RW, Lips KR (2014) Unexpected rarity of the pathogen *Batrachochytrium dendrobatidis* in Appalachian Plethodon salamanders: 1957-2011. *PLoS ONE* 9 e103728

Muletz-Wolz CR, DiRenzo GV, Yarwood SA, Campbell Grant EH, Fleischer RC, Lips KR (2017) Antifungal bacteria on woodland salamander skin exhibit high taxonomic diversity and geographic variability. *Applied and Environmental Microbiology* 83 e00186-17

Murphy PJ, St-Hilaire S, Bruer S, Corn PS, Peterson CR (2009) Distribution and pathogenicity of *Batrachochytrium dendrobatidis* in boreal toads from the Grand Teton Area of Western Wyoming. *EcoHealth* 6:109-120

Murray KA, Skerratt LF, Garland S, Kriticos D, McCallum H (2013) Whether the weather drives patterns of endemic amphibian chytridiomycosis: A pathogen proliferation approach. *PLoS ONE* 8 e61061

Murray KA, Skerratt LF, Speare R, McCallum H (2009) Impact and dynamics of disease in species threatened by the amphibian chytrid fungus *Batrachochytrium dendrobatidis*. *Conservation Biology* 23:1242-1252

Murrieta-Galindo R, Parra-Olea G, Gonzalez-Romero A, Lopez-Barrera F, Vredenburg VT (2014) Detection of *Batrachochytrium dendrobatidis* in amphibians inhabiting cloud forests and coffee agroecosystems in Central Veracruz, Mexico. *European Journal of Wildlife Research* 60:431-439

Muths E (2003) Home range and movements of boreal toads in undisturbed habitat. *Copeia* 1:160-165

Muths E, Corn P, Pessier A, Green D (2003) Evidence for disease-related amphibian decline in Colorado. *Biological Conservation* 110:357-365

Muths E, Pilliod DS, Livo LJ (2008) Distribution and environmental limitations of an amphibian pathogen in the Rocky Mountains, USA. *Biological Conservation* 141:1484-1492.

Nair A, Daniel O, Gopalan SV, George S, Kumar KS, Merilä, J, Teracher AGF (2011) Infectious disease screening of Indirana frogs from the Western Ghats biodiversity hotspot. *Herpetological Review* 42:554-557

Narayan E, Molinia F, Hero J-M, 2011. Absence of invasive chytrid fungus (*Batrachochytrium dendrobatidis*) in native Fijian ground frog (*Platymantis vitiana*) populations on Viwa-Tailevu, Fiji islands. *Acta Herpetologica* 6:261-266

Narayan EJ, Graham C, McCallum H, Hero J-M (2014) Over-wintering tadpoles of *Mixophyes fasciolatus* act as reservoir host for *Batrachochytrium dendrobatidis*. *PLoS ONE* 9 e92499

Nava-González BA, Suazo-Ortuño I, Parra-Olea G, López-Toledo L, Alvarado-Díaz J (2019) *Batrachochytrium dendrobatidis* infection in amphibians from a high elevation habitat in the Trans-Mexican Volcanic Belt. *Aquatic Ecology* 54:75-87

Navarro-Lozano A, Sánchez-Domene D, Rossa-Feres DC, Bosch J, Sawaya RJ (2018) Are oral deformities in tadpoles accurate indicators of anuran chytridiomycosis? *PLOS ONE* 13 e0190955

Newman JC, Mota JL, Hardman RH, Dillman JW, Barrett K (2019) Pathogen detection in green salamanders (*Aneides aeneus*) in South Carolina, USA. *Herpetological Review* 50:503–505

Nieto NC, Camann MA, Foley JE, Reiss JO (2007) Disease associated with integumentary and cloacal parasites in tadpoles of Northern red-legged frog *Rana aurora aurora*. *Diseases of Aquatic Organisms* 78:61-71

North S, Alford RA (2008) Infection intensity and sampling locality affect *Batrachochytrium dendrobatidis* distribution among body regions on green-eyed tree frogs *Litoria genimaculata*. *Diseases of Aquatic Organisms* 81:177-188

Ocock JF, Rowley JJL, Penman TD, Rayner TS, Kingsford RT (2013) Amphibian chytrid prevalence in an amphibian community in arid australia. *EcoHealth* 10:77-81

Ohst T, Graeser Y, Plötner J (2013) *Batrachochytrium dendrobatidis* in Germany: Distribution, prevalences and prediction of high risk areas. *Diseases of Aquatic Organisms* 107:49-59

Oficialdegui FJ, Sánchez MI, Monsalve-Carcaño C, Boyero L, Bosch J (2019) The invasive red swamp crayfish (*Procambarus clarkii*) increases infection of the amphibian chytrid fungus (*Batrachochytrium dendrobatidis*). *Biological Invasions* 21:3221-3231

Oliveira De Queiroz Carnaval AC, Puschendorf R, Peixoto OL, Verdade VK, Rodrigues MT (2006) Amphibian chytrid fungus broadly distributed in the Brazilian Atlantic Rain Forest. *EcoHealth* 3:41-48

Ouellet M, Mikaelian I, Pauli B, Rodrigue J, Green D (2005) Historical evidence of widespread chytrid infection in North American amphibian populations. *Conservation Biology* 19:1431-1440

Padgett-Flohr GE, Goble ME (2007) Evaluation of tadpole mouthpart depigmentation as a diagnostic test for infection by *Batrachochytrium dendrobatidis* for four California anurans. *Journal of Wildlife Diseases* 43:690-699

Padgett-Flohr GE, Hopkins RL, II (2010) Landscape epidemiology of *Batrachochytrium dendrobatidis* in Central California. *Ecography* 33:688-697

Padgett-Flohr GE, Hopkins RL, II (2009) *Batrachochytrium dendrobatidis*, a novel pathogen approaching endemism in Central California. *Diseases of Aquatic Organisms* 83:1-9

Pasmans F, Muijsers M, Maes S, Van Rooij P, Brutyn M, Ducatelle R, Haesebrouck F, Martel A (2010) Chytridiomycosis related mortality in a midwife toad (*Alytes obstetricans)* in Belgium. *Tijdschrift Voor Diergeneeskunde*. 79:460-462

Pasmans F, Van Rooij P, Blooi M, Tessa G, Bogaerts S, Sotgiu G, Garner TWJ, Fisher MC, Schmidt BR, Wöltjes T, Beukema W, Bovero S, Adriaensen C, Oneto F, Ottonello D, Martel A, Salvidio S (2013) Resistance to chytridiomycosis in European plethodontid salamanders of the genus Speleomantes. *PLoS ONE* 8 e63639

Patillo BE, Parris MJ (2016) High prevalence of *Batrachochytrium dendrobatidis* in *Notophthalmus viridescens* in the Ozark National Forest and Harold E. Alexander Wildlife Management Area Arkansas, USA. *Herpetological Review* 47:210-211

Patrelle C, Miaud C, Cristina N, Kulberg P, Merilä, J (2012) Chytrid fungus screening in a population of common frogs from Northern Finland. *Herpetological Review* 43:422-425

Pauza MD, Driessen MM, Skerratt LF (2010) Distribution and risk factors for spread of amphibian chytrid fungus *Batrachochytrium dendrobatidis* in the Tasmanian Wilderness World Heritage Area, Australia. *Diseases of Aquatic Organisms* 92:193-199

Pearl CA, Bowerman J, Adams MJ, Chelgren ND (2009) Widespread occurrence of the chytrid fungus *Batrachochytrium dendrobatidis* on Oregon spotted frogs (*Rana pretiosa*). *EcoHealth* 6:209-218

Pearl CA, Bull EL, Green DE, Bowerman J, Adams MJ, Hyatt A, Wente WH (2007) Occurrence of the amphibian pathogen *Batrachochytrium dendrobatidis* in the Pacific Northwest. *Journal of Herpetology* 41:145-149

Penner J, Adum GB, McElroy MT, Doherty-Bone T, Hirschfeld M, Sandberger L, Weldon C, Cunningham AA, Ohst T, Wombwell E, Portik DM, Reid D, Hillers A, Ofori-Boateng C, Oduro W, Ploetner J, Ohler A, Leache AD, Roedel M-O (2013) West Africa - A safe haven for frogs? A sub-continental assessment of the Chytrid Fungus (*Batrachochytrium dendrobatidis*). *PLoS ONE 8 e56236*

Paetow LJ, Pauli BD, McLaughlin DJ, Bidulka J, Marcogliese DJ (2011) First detection of *Ranavirus* in *Lithobates pipiens* in Quebec. *Herpetological Review* 42:211-214

Peralta-García A, Adams A, Briggs C, Galina-Tessaro P, Valdez-Villavicencio J, Hollingsworth B, Shaffer H, Fisher R (2018) Occurrence of *Batrachochytrium dendrobatidis* in anurans of the Mediterranean region of Baja California Mexico. *Diseases of Aquatic Organisms* 127:193-200

Perez R, Richards-Zawacki CL, Krohn AR, Robak M, Griffith EJ, Ross H, Gratwicke B, Ibanez R, Voyles J (2014) Field surveys in Western Panama indicate populations of *Atelopus varius* frogs

are persisting in regions where *Batrachochytrium dendrobatidis* is now enzootic. *Amphibian, Reptile Conservation* 8 e85
Perl RGB, Gafny S, Malka Y, Renan S, Woodhams DC, Rollins-Smith L, Pask JD, Bletz MC, Geffen E & Vences M (2017) Natural history and conservation of the rediscovered hula painted frog, *Latonia nigriventer*. *Contributions to Zoology* 86:11-37
Petersen CE, Lovich RE, Phillips CA, Dreslik MJ, Lannoo MJ (2016) Prevalence and seasonality of the amphibian chytrid fungus *Batrachochytrium dendrobatidis* along widely separated longitudes across the United States. *EcoHealth* 13:368-382
Peterson AC, McKenzie VJ (2014) Investigating differences across host species and scales to explain the distribution of the amphibian pathogen *Batrachochytrium dendrobatidis*. *PLoS ONE* 9 e107441
Peterson JD, Wood MB, Hopkins WA, Unrine JM, Mendonca MT (2007) Prevalence of *Batrachochytrium dendrobatidis* in American bullfrog and Southern leopard frog larvae from wetlands on the Savannah River Site South Carolina. *Journal of Wildlife Diseases* 43:450-460
Phillips CA, Wesslund NA, MacAllister IE (2014) Occurrence of the Chytrid Fungus *Batrachochytrium dendrobatidis* in amphibians in Illinois, USA. *Herpetological Review* 45:238-240
Phillott AD, Grogan LF, Cashins SD, Mcdonald KR, Berger L, Skerratt LF (2013) Chytridiomycosis and seasonal mortality of tropical stream-associated frogs 15 years after introduction of *Batrachochytrium dendrobatidis*. *Conservation Biology* 27:1058-1068
Picco AM, Collins JP (2007) Fungal and viral pathogen occurrence in Costa Rican amphibians. *Journal of Herpetology* 41:746-749
Pilliod DS, Muths E, Scherer RD, Bartelt PE, Corn PS, Hossack BR, Lambert BA, Mccaffery R, Gaughan C (2010) Effects of amphibian chytrid fungus on individual survival probability in wild boreal toads. *Conservation Biology* 24:1259-1267
Piovia-Scott J, Pope KL, Lawler SP, Cole EM, Foley JE (2011) Factors related to the distribution and prevalence of the fungal pathogen *Batrachochytrium dendrobatidis* in *Rana cascadae* and other amphibians in the Klamath Mountains. *Biological Conservation* 144:2913-2921
Pope KL, Wengert GM, Foley JE, Ashton DT, Botzler RG, Grp EA (2016) Citizen scientists monitor a deadly fungus threatening amphibian communities in Northern Coastal California, USA. *Journal of Wildlife Diseases* 52:516-523
Preuss JF, Lambertini C, Leite D. Da S, Toledo LF, Lucas EM (2016) Crossing the threshold: An amphibian assemblage highly infected with *Batrachochytrium dendrobatidis* in the Southern Brazilian Atlantic Forest. *Studies on neotropical fauna and environment* 51:68-77
Puschendorf R, Bolanos F, Chaves G (2006a) The amphibian chytrid fungus along an altitudinal transect before the first reported declines in Costa Rica. *Biological Conservation* 132:136-142
Puschendorf R, Castaneda F, McCranie JR (2006b) Chytridiomycosis in wild frogs from Pico Bonito National Park, Honduras. *EcoHealth* 3:178-181
Puschendorf R, Hoskin CJ, Cashins SD, McDonald K, Skerratt LF, Vanderwal J, Alford RA (2011) Environmental refuge from disease-driven amphibian extinction. *Conservation Biology* 25:956-964
Rachowicz LJ (2002) Mouthpart pigmentation in *Rana muscosa* tadpoles: Seasonal changes without chytridiomycosis. Herpetological review 33:263-265
Rachowicz LJ, Briggs CJ (2007) Quantifying the Disease Transmission Function: Effects of Density on *Batrachochytrium dendrobatidis* Transmission in the Mountain Yellow-legged Frog *Rana muscosa*. *Journal of Animal Ecology* 76:711-721
Rachowicz LJ, Knapp RA, Morgan JAT, Stice MJ, Vredenburg VT, Parker JM, Briggs CJ (2006) Emerging Infectious Disease as a Proximate Cause of Amphibian Mass Mortality. *Ecology* 87:1671-1683

Ramesh R, Lord A, Griffis-Kyle K, Perry G, Hamilton D, Silva S (2013) Amphibian Populations in Brazos River Basin Texas Show No Evidence of *Bd* Infection. *Herpetological Review* 44: 461-464

Raverty S, Reynolds T (2001) Cutaneous chytridiomycosis in dwarf aquatic frogs (*Hymenochirus böttgeri*) originating from Southeast Asia and in a Western toad (*Bufo boreas*) from Northeastern British Columbia. *Canadian Veterinary Journal* 42:385-386

Rebollar EA, Hughey MC, Harris RN, Domangue RJ, Medina D, Ibanez R, Belden LK (2014) The Lethal fungus *Batrachochytrium dendrobatidis* is present in lowland tropical forests of far Eastern Panama. *PLoS ONE* 9 e95484

Rebollar EA, Hughey MC, Medina D, Harris RN, Ibanez R, Belden LK (2016) Skin bacterial diversity of Panamanian frogs is associated with host susceptibility and presence of *Batrachochytrium dendrobatidis*. *Multidisciplinary Journal of Microbial Ecology* 10:1682-1695

Reeder NMM, Pessier AP, Vredenburg VT (2012) a Reservoir species for the emerging amphibian pathogen *Batrachochytrium dendrobatidis* thrives in a landscape decimated by disease. *PLoS ONE* 7 e33567

Reeves RA, Pierce CL, Vandever MW, Muths E, Smalling KL (2017) Amphibians, pesticides, and the amphibian chytrid fungus in restored wetlands in agricultural landscapes. Herpetological *Conservation and Biology* 12:68-77

Rendle M, Tapley B, Perkins M, Bittencourt-Silva G, Gower DJ, Wilkinson M (2015) Itraconazole Treatment of *Batrachochytrium dendrobatidis* (*Bd*) Infection in captive caecilians (Amphibia: Gymnophiona) and the first case of *Bd* in a wild neotropical caecilian. *Journal of Zoo and Aquarium Research* 3:137-140

Regester KJ, Simpson H, Chapman EJ, Petokas PJ (2012) Occurrence of the fungal pathogen *Batrachochytrium dendrobatidis* among eastern hellbender populations (*Cryptobranchus a. alleganiensis*) within the Allegheny-Ohio and Susquehanna River drainages Pennsylvania, USA. *Herpetological Review* 43:90-93

Reshetnikov AN, Chestnut T, Brunner JL, Charles K, Nebergall EE, Olson DH (2014) Detection of the Emerging Amphibian Pathogens *Batrachochytrium dendrobatidis* and *Ranavirus* in Russia. *Diseases of Aquatic Organisms* 110:235-240

Retallick R, McCallum H, Speare R (2004) Endemic infection of the amphibian chytrid fungus in a frog community post-decline. *PLoS Biol*. 2:1965-1971

Ribeiro JW, Siqueira T, DiRenzo GV, Lambertini C, Lyra ML, Toledo LF, Haddad CFB, Becker CG (2020) Assessing amphibian disease risk across tropical streams while accounting for imperfect pathogen detection. *Oecologia* https://doi.org/10.1007/s00442-020-04646-4

Richards CL, Zellmer AJ, Martens LM (2008) *Batrachochytrium dendrobatidis* not detected in *Oophaga pumilio* on Bastimentos Island, Panama. *Herpetological Review* 39:200-202

Richards-Hrdlicka KL (2012) Extracting the Amphibian Chytrid Fungus from formalin-fixed specimens. *Methods in Ecology and Evolution* 3:842-849

Richards-Hrdlicka KL, Richardson JL, Mohabir L (2013) First survey for the amphibian chytrid fungus *Batrachochytrium dendrobatidis* in Connecticut (USA) finds widespread prevalence. *Diseases of Aquatic Organisms* 102:169-180

Richardson JML, Govindarajulu P, Anholt BR (2014) Distribution of the disease pathogen *Batrachochytrium dendrobatidis* in non-epidemic amphibian communities of Western Canada. Ecography 37:883-893

Richards-Zawacki CL (2010) Thermoregulatory behaviour affects prevalence of chytrid fungal infection in a wild population of Panamanian golden frogs. *Proceedings of the Royal Society B: Biological Sciences* 277:519-528

Richter SC, Drayer AN, Strong JR, Kross CS, Miller DL, Gray MJ (2013) High prevalence of *Ranavirus* infection in permanent constructed wetlands in Eastern Kentucky, USA. *Herpetological Review* 44:464-466

Riley K, Berry OF, Roberts JD (2013) Do global models predicting environmental suitability for the amphibian fungus *Batrachochytrium dendrobatidis* have local value to conservation managers? *Journal of Applied Ecology* 50:713-720

Rimer RL, Briggler JT (2010) Occurrence of the amphibian chytrid fungus (*Batrachochytrium dendrobatidis*) in Ozark Caves Missouri, USA. *Herpetological Review* 41:175-177

Rios-sotelo G, Figueroa-Valenzuela R, Vredenburg VT (2018) Retrospective survey reveals extreme rarity of amphibian fungal pathogen *Batrachochytrium dendrobatidis* in Japanese amphibians from 1890–1990s. *Herpetological Review* 49:247-252

Rivera B, Cook K, Andrews K, Atkinson MS, Savage AE (2019) Pathogen dynamics in an invasive frog compared to native species. *Ecohealth* 16:222-234

Rizkalla CE (2010) Increasing detections of *Batrachochytrium dendrobatidis* in Central Florida, USA. *Herpetological Review* 41:180-181

Robinson CW, McNulty SA, Titus VR (2018) No safe space: Prevalence and distribution of *Batrachochytrium dendrobatidis* in amphibians in a highly-protected landscape. *Herpetological Conservation and Biology* 13:373-382

Rodriguez D, Becker CG, Pupin NC, Haddad CFB, Zamudio KR (2014) Long-term endemism of two highly divergent lineages of the amphibian-killing fungus in the Atlantic Forest of Brazil. *Molecular Ecology* 23:774-787

Rodriguez LO, Catenazzi A (2017) Four new species of terrestrial-breeding frogs of the genus Phrynopus (Anura: Terrarana: Craugastoridae) from Río Abiseo National Park Peru. *Zootaxa* 4273:381-406

Rodriguez-Brenes S, Rodriguez D, Ibanez R, Ryan MJ (2016) Spread of amphibian chytrid fungus across lowland populations of tungara frogs in Panama. *PLoS ONE* 11 e0155745

Rodriguez-Contreras A, Senaris JC, Lampo M, Rivero R (2008) Rediscovery of *Atelopus cruciger* (Anura : Bufonidae): Current status in the Cordillera de la Costa, Venezuela. *Oryx* 42:301-304

Rollins-Smith LA, Reinert LK, Burrowes PA (2015) Coqui frogs persist with the deadly chytrid fungus despite a lack of defensive antimicrobial peptides. *Diseases of Aquatic Organisms* 113:81-83

Rosa GM, Anza I, Moreira PL, Conde J, Martins F, Fisher MC, Bosch J (2013) Evidence of chytrid-mediated population declines in common midwife toad in Serra da Estrela Portugal. *Animal Conservation* 16:306-315

Rosa GM, Sabino-Pinto J, Laurentino TG, Martel A, Pasmans F, Rebelo R, Griffiths RA, Stöhr AC, Marschang RE, Price SJ, Garner TWJ, Bosch J (2017) Impact of asynchronous emergence of two lethal pathogens on amphibian assemblages. *Scientific Reports* 7 43260

Ross L, Wright M, Wiskirchen K, Grace J, Lennon C, Mantooth J, Schneider D, Hudman PS, Kelrick MI, Montgomery CE (2014) Prevalence of batrochochytrium *dendrobatidis* in three frog species of the Bighorn National Forest Wyoming, USA. *Herpetological Review* 45:615- 616

Rothermel BB, Miller DL, Travis ER, McGuire JLG, Jensen JB, Yabsley MJ (2016) Disease dynamics of red-spotted newts and their anuran prey in a montane pond community. *Diseases of Aquatic Organisms* 118:113-127

Rothermel BB, Travis ER, Miller DL, Hill RL, McGuire JL, Yabsley MJ (2013) High occupancy of stream salamanders despite high *Ranavirus* prevalence in a Southern Appalachians watershed. *EcoHealth* 10:184-189

Rothermel BB, Walls SC, Mitchell JC, Dodd CK, Jr Irwin LK, Green DE, Vazquez VM, Petranka JW, Stevenson DJ (2008) Widespread occurrence of the amphibian chytrid fungus *Batrachochytrium dendrobatidis* in the Southeastern, USA. *Diseases of Aquatic Organisms* 82:3-18

Roznik EA, Sapsford SJ, Pike DA, Schwarzkopf L, Alford RA (2015a) Natural disturbance reduces disease risk in endangered rainforest frog populations. Scientific Reports 5.

Roznik EA, Sapsford SJ, Pike DA, Schwarzkopf L, Alford RA (2015b) Condition-dependent reproductive effort in frogs infected by a widespread pathogen. *Proceedings of the Royal Society B: Biological Sciences* 282:20150694

Rowley JJL, Skerratt LF, Alford RA, Campbell R (2007) Retreat sites of rain forest stream frogs are not a reservoir for *Batrachochytrium dendrobatidis* in Northern Queensland, Australia. *Diseases of Aquatic Organisms* 74:7-12

Rubio A, Kupferberg S, Vargas García V, Ttito A, Shepack A, Catenazzi A (2018) Widespread occurrence of the antifungal cutaneous bacterium J*anthinobacterium lividum* on Andean water frogs threatened by fungal disease. *Diseases of Aquatic Organisms* 131:233-238

Ruggeri J, Longo AV, Gaiarsa MP, Alencar LRV, Lambertini C, Leite DS, Carvalho-e-Silva SP, Zamudio KR, Toledo LF, Martins M (2015) Seasonal variation in population abundance and chytrid infection in stream-dwelling frogs of the Brazilian Atlantic Forest. *PLoS ONE* 10 e0130554

Ruiz A, Rueda-Almonacid JV (2008) *Batrachochytrium dendrobatidis* and chytridiomycosis in anuran amphibians of Colombia. *EcoHealth* 5:27-33

Russell DM, Goldberg CS, Waits LP, Rosenblum EB (2010) *Batrachochytrium dendrobatidis* infection dynamics in the Columbia spotted frog *Rana Luteiventris* in North Idaho, USA. *Diseases of Aquatic Organisms* 92:223-230

Ryan MJ, Latella IM, Painter CW, Giermakowski JT, Christman BL, Jennings RD, Voyles JL (2014) First record of *Batrachochytrium dendrobatidis* in the Arizona toad (*Anaxyrus microscaphus*) in Southwestern New Mexico, USA. *Herpetological Review* 45:616-618

Sacerdote-Velat A, Manjerovic MB, Santymire R (2016) Preliminary survey of *Batrachochytrium dendrobatidis* in the Chicago region of Illinois, USA. *Herpetological Review* 47:57-58

Sadinski W, Roth M, Treleven S, Theyerl J, Dummer P (2010) Detection of the chitrid fungus *Batrachochytrium dendrobatidis* on recently metamorphosed amphibians in the North-Central United States. *Herpetological Review* 41:170-175

Saenz D, Kavanagh BT, Kwiatkowski MA (2010) *Batrachochytrium dendrobatidis* detected in amphibians from national forests in Eastern Texas, USA. *Herpetological Review* 41:47-49

Saenz D, Hall TL, Kwiatkowski MA (2015) Effects of urbanization on the occurrence of *Batrachochytrium dendrobatidis*: Do urban environments provide refuge from the amphibian chytrid fungus? Urban Ecosystems 18:333-340

Sainsbury AW, Yu-Mei R, Ågren E, Vaughan-Higgins RJ, Mcgill IS, Molenaar F, Peniche G, Foster J (2016) Disease risk analysis and post-release health surveillance for a reintroduction programme: The pool frog *Pelophylax lessonae*. *Transboundary and Emerging Diseases* 64:1530-1548

Salla RF, Rizzi-Possignolo GM, Oliveira CR, Lambertini C, Franco-Belussi L, Leite DS, Silva-Zacarin ECM, A*bd*alla FC, Jenkinson TS, Toledo LF, Jones-Costa M (2018) Novel Findings on the Impact of Chytridiomycosis on the Cardiac Function of anurans: Sensitive vs Tolerant Species. *PeerJ* 6 e5891.

Sanchez D, Chacon-Ortiz A, Leon F, Han BA, Lampo M (2008) Widespread occurrence of an emerging pathogen in amphibian communities of the Venezuelan Andes. *Biological Conservation* 141:2898-2905

Sapsford SJ, Alford RA, Schwarzkopf L (2013) Elevation, temperature, and aquatic connectivity all influence the infection dynamics of the amphibian chytrid fungus in adult frogs. *PLoS ONE* 8 e82425

Sapsford SJ, Voordouw MJ, Alford RA, Schwarzkopf L (2015) Infection dynamics in frog populations with different histories of decline caused by a deadly disease. *Oecologia* 179:1099-1110

Savage AE, Becker CG, Zamudio KR (2015) Linking Genetic and environmental factors in amphibian disease risk. *Evolutionary Applications* 8:560-572

Savage AE, Grismer LL, Anuar S, Onn CK, Grismer JL, Quah E, Muin MA, Ahmad N, Lenker M, Zamudio KR (2011a) First record of *Batrachochytrium dendrobatidis* infecting four frog families from peninsular Malaysia. *EcoHealth* 8:121-128

Savage AE, Sredl MJ, Zamudio KR (2011b) Disease dynamics vary spatially and temporally in a North American amphibian. *Biological Conservation* 144:1910-1915

Savage AE, Zamudio KR (2016) Adaptive tolerance to a pathogenic fungus drives major histocompatibility complex evolution in natural amphibian populations. *Proceedings of the Royal Society B: Biological Sciences* 283:20153115

Scalera R, Adams MJ, Galvan SK (2008) Occurrence of *Batrachochytrium dendrobatidis* in amphibian populations in Denmark. *Herpetological Review* 39:199-200

Scheele B. C, Driscoll DA, Fischer J, Fletcher AW, Hanspach J, Voros J, Hartel T (2015) Landscape context influences chytrid fungus distribution in an endangered European amphibian. *Animal Conservation* 18:480-488

Scheele BC, Guarino F, Osborne W, Hunter DA, Skerratt LF, Driscoll DA (2014) Decline and re-expansion of an amphibian with high prevalence of chytrid fungus. *Biological Conservation* 170:86-91

Scheele Ben C, Hunter DA, Skerratt LF, Brannelly LA, Driscoll DA (2015) Low impact of chytridiomycosis on frog recruitment enables persistence in refuges despite high adult mortality. *Biological Conservation* 182:36-43

Scheele BC, Hunter DA, Brannelly LA, Skerratt LF, Driscoll DA (2016) Reservoir-host amplification of disease impact in an endangered amphibian. *Conservation Biology* 31:592- 600

Scheele BC, Hunter DA, Banks SC, Pierson JC, Skerratt LF, Webb R, Driscoll DA (2016) High adult mortality in disease-challenged frog populations increases vulnerability to drought. *Journal of Animal Ecology* 85:1453-1460

Schlaepfer MA, Sredl MJ, Rosen PC, Ryan MJ (2007) High prevalence of *Batrachochytrium dendrobatidis* in wild populations of lowland leopard frogs *Rana yavapaiensis* in Arizona. *EcoHealth* 4:421-427

Schock DM, Ruthig GR, Collins JP, Kutz SJ, Carriere S, Gau RJ, Veitch AM, Larter NC, Tate DP, Guthrie G, Allaire DG, Popko RA (2010) Amphibian chytrid fungus and *Ranavirus*es in the Northwest territories Canada. *Diseases of Aquatic Organisms* 92:231-240

Seeley KE, D'Angelo M, Gowins C, Greathouse J (2016) Prevalence of *Batrachochytrium dendrobatidis* in Eastern hellbender (*Cryptobranchus alleganiensis*) populations in West Virginia, USA. *Journal of Wildlife Diseases* 52:391-394

Seimon TA, Seimon A, Daszak P, Halloy SRP, Schloegel LM, Aguilar CA, Sowell P, Hyatt AD, Konecky B, E Simmons J (2007) Upward range extension of Andean anurans and chytridiomycosis to extreme elevations in response to tropical deglaciation. *Global Change Biology* 13:288-299

Seimon TA, Ayebare S, Sekisambu R, Muhindo E, Mitamba G, Greenbaum E, Menegon M, Pupin F, McAloose D, Ammazzalorso A, Meirte D, Lukwago W, Behangana M, Seimon A, Plumptre AJ (2015) Assessing the Threat of Amphibian Chytrid Fungus in the Albertine Rift: Past Present and Future. *PLoS ONE* 10 e0145841

Seimon TA, Seimon A, Yager K, Reider K, Delgado A, Sowell P, Tupayachi A, Konecky B, McAloose D, Halloy S (2017) Long-term monitoring of tropical alpine habitat change Andean Anurans and chytrid fungus in the Cordillera Vilcanota Peru: Results from a decade of study. *Ecology and Evolution* 7:1527-1540

Sette CM, Vredenburg VT, Zink AG (2015) Reconstructing historical and contemporary disease dynamics: A case study using the California slender salamander. *Biological Conservation* 192:20-29

Sigafus BH, Schwalbe CR, Hossack BR, Muths E (2014) Prevalence of the amphibian chytrid fungus (*Batrachochytrium dendrobatidis*) at Buenos Aires National Wildlife Refuge Arizona, USA. *Herpetological Review* 45:41-42

Simpkins C, Hero J-M, Van Sluys M (2010) Detecting the Western limits for *Batrachochytrium dendrobatidis* in Southeastern Queensland, Australia. *Herpetological Review* 41:454-456

Simpkins CA, Kriger K, Hero JM (2017) Prevalence of *Batrachochytrium dendrobatidis* on amphibians from low pH, oligotrophic waterbodies. *Herpetological Review* 48:775-776

Skerratt LF, McDonald KR, Hines HB, Berger L, Mendez D, Phillott AD, Cashins SD, Murray KA, Speare R (2010) Application of the survey protocol for chytridiomycosis to Queensland, Australia. *Diseases of Aquatic Organisms* 92:117-129

Skerratt LF, Mendez D, McDonald KR, Garland S, Livingstone J, Berger L, Speare R (2011) Validation of diagnostic tests in wildlife: The case of chytridiomycosis in wild amphibians. *Journal of Herpetology* 45:444-450

Smith SN, Watters JL, Ellsworth ED, Davis DR, Siler CD (2019) Assessment of *Batrachochytrium dendrobatidis* and *ranavirus* among wild amphibians from four Philippine Islands. *Herpetological Review* 50:729–734

Smith KG, Weldon C, Conradie W, Du Preez LH (2007) Relationships among size, development, and *Batrachochytrium dendrobatidis* infection in African tadpoles. *Diseases of Aquatic Organisms* 74:159-164

Smith TC, Picco AM, Knapp R (2017) *Ranavirus*es Infect mountain yellow-legged frogs (*Rana Muscosa* and *Rana sierrae*) threatened by *Batrachochytrium dendrobatidis*. *Herpetological Conservation and Biology* 12:149-159

Solis R, Lobos G, Walker SF, Fisher M, Bosch J (2010) Presence of *Batrachochytrium dendrobatidis* in feral populations of *Xenopus laevis* in Chile. *Biological Invasions* 12:1641-1646

Solis R, Penna M, De La Riva I, Fisher MC, Bosch J (2015) Presence of *Batrachochytrium dendrobatidis* in anurans from the Andes Highlands of Northern Chile. *Herpetological Journal* 25:55-59

Sonn JM, Utz RM, Richards-Zawacki CL (2019) Effects of latitudinal seasonal and daily temperature variations on chytrid fungal infections in a North American Frog. *Ecosphere* 10 e02892

Soto-Azat C, Valenzuela-Sanchez A, Clarke BT, Busse K, Carlos Ortiz J, Barrientos C, Cunningham AA (2013) Is chytridiomycosis driving Darwin's frogs to extinction? *PLoS ONE* 8 e79862

Soto-Azat C, Peñafiel-Ricaurte A, Price SJ, Sallaberry-Pincheira N, García MP, Alvarado-Rybak M, Cunningham AA (2016) *Xenopus laevis* and emerging amphibian pathogens in Chile. *Ecohealth* 13:775-783

Souza MJ, Gray MJ, Colclough P, Miller DL (2012) Prevalence of infection by *Batrachochytrium dendrobatidis* and *ranavirus* in Eastern hellbenders (*Cryptobranchus alleganiensis alleganiensis*) in Eastern Tennessee. *Journal of Wildlife Diseases* 48:560-566

Spaulding SH, Cox JJ, Maigret TA, Drayer AN, Richards JM, Treanor J (2018) Low-level *Batrachochytrium dendrobatidis* detection persists in plethodontid salamanders following timber harvest in Kentucky, USA. *Herpetological Review* 49:258–262

Spitzen-van Der Sluijs A, Martel A, Asselberghs J, Bales EK, Beukema W, Bletz MC, Dalbeck L, Goverse E, Kerres A, Kinet T, Kirst K, Laudelout A, Da Fonte LFM, Nöllert A, Ohlhoff D, Sabino-Pinto J, Schmidt BR, Speybroeck J, Spikmans F, Steinfartz S, Veith M, Vences M, Wagner N, Pasmans F, Lötters S (2016) Expanding distribution of lethal amphibian fungus *Batrachochytrium Salamandrivorans* in Europe. *Emerging Infectious Diseases* 22:1286-1288

Spitzen-Van Der Sluijs A, Martel A, Hallmann CA, Bosman W, Garner TWJ, Van Rooij P, Jooris R, Haesebrouck F, Pasmans F (2014) Environmental determinants of recent endemism of *Batrachochytrium dendrobatidis* infections in amphibian assemblages in the absence of disease outbreaks. *Conservation Biology* 28:1302-1311

Spitzen-van der Sluijs A, Canessa S, Martel A, Pasmans F (2017) Fragile coexistence of a global chytrid pathogen with amphibian populations is mediated by environment and demography. *Proceedings of the Royal Society B: Biological Sciences* 284 20171444

Standish I, Leis E, Schmitz N, Credico J, Erickson S, Bailey J, Kerby J, Phillips K, Lewis T (2018) Optimizing validating and field testing a multiplex qPCR for the detection of amphibian Pathogens. *Diseases of Aquatic Organisms* 129:1-13

Stark T, Laurijssens C, Weterings M, Martel A, Köhler G, Pasmans F (2017) Prevalence of *Batrachochytrium dendrobatidis* in a Nicaraguan Micro-endemic neotropical salamander B*olitoglossa mombachoensis*. *Amphibia-Reptilia* 38:102-107

Steiner SL, Lehtinen RM (2008) Occurrence of the amphibian pathogen *Batrachochytrium dendrobatidis* in Blanchard's Cricket Frog (*Acris crepitans blanchardi*) in the US. Midwest. *Herpetological Review* 39:193-196

St-Amour V, Garner TWJ, Schulte-Hostedde AI, Lesbarreres D (2010) Effects of two amphibian pathogens on the developmental stability of green frogs. *Conservation Biology* 24:788-794

Stockwell MP, Bower DS, Bainbridge L, Clulow J, Mahony MJ (2015a) Island provides a pathogen refuge within climatically suitable area. *Biodiversity and Conservation* 24:2583-2592

Stockwell MP, Bower DS, Clulow J, Mahony MJ (2016) The role of non-declining amphibian species as alternative hosts for *Batrachochytrium dendrobatidis* in an amphibian community. *Wildlife Research* 43:341-347

Stutz WE, Blaustein AR, Briggs CJ, Hoverman JT, Rohr JR, Johnson PTJ (2017) Using multi-response models to investigate pathogen coinfections across scales: Insights from emerging diseases of amphibians. *Methods in. Ecology and Evolution* 9:1109-1120

Suriyamongkol T, Villamizar-Gomez A, Forstner MRJ, Mali I (2019) Detection of *Batrachochytrium dendrobatidis* in Eastern New Mexico, USA. *Herpetological Review* 50:300-303

Swei A, Rowley JJL, Rödder D, Diesmos MLL, Diesmos AC, Briggs CJ, Brown R, Cao TT, Cheng TL, Chong RA, Han B, Hero J-M, Hoang HD, Kusrini MD, Le DTT, McGuire JA, Meegaskumbura M, Min M-S, Mulcahy DG, Neang T, Phimmachak S, Rao D-Q, Reeder NM, Schoville SD, Sivongxay N, Srei N, Stöck M, Stuart BL, Torres LS, Tran DTA, Tunstall TS, Vieites D, Vredenburg VT (2011) Is chytridiomycosis an emerging infectious disease in Asia? *PLoS ONE* 6 e23179

Symonds EP, Hines HB, Bird PS, Morton JM, Mills PC (2007) Surveillance for *Batrachochytrium dendrobatidis* using Mixophyes (Anura : Myobatrachidae) larvae. *Journal of Wildlife Diseases* 43:48-60

Sztatecsny M, Glaser F (2011) From the Eastern lowlands to the Western mountains: First records of the chytrid fungus *Batrachochytrium dendrobatidis* in wild amphibian populations from Austria. *Herpetological Journal* 21:87-90

Sweeney R (2016) First detection of the amphibian chytrid fungus (*Batrachochytrium dendrobatidis*) in St. Vincent and the Grenadines. *Herpetological Review* 47:212-214

Talley BL, Lips KR, Ballard SR (2011) *Batrachochytrium dendrobatidis* in *Siren intermedia* in Illinois, USA. *Herpetological Review* 42:216-217

Talley BL, Muletz CR, Vredenburg VT, Fleischer RC, Lips KR (2015) A century of *Batrachochytrium dendrobatidis* in Illinois Amphibians (1888-1989). *Biological Conservation* 182:254-261

Tarrant J, Cilliers D, Du Preez LH, Weldon C (2013) Spatial assessment of amphibian chytrid fungus (*Batrachochytrium dendrobatidis*) in South Africa Confirms endemic and widespread infection. *PLoS ONE* 8 e69591

Terrell VCK, Engbrecht NJ, Pessier AP, Lannoo MJ (2014) Drought reduces chytrid fungus (*Batrachochytrium dendrobatidis*) infection intensity and mortality but not prevalence in adult crawfish frogs (*Lithobates areolatus*). *Journal of Wildlife Diseases* 50:56-62

Tatarian P, Tatarian G (2010) Chytrid infection of *Rana draytonii* in the Sierra Nevada California, USA. *Herpetological Review* 41:325-327

Thompson LM, Pugh B, McDonald LA, Estrada A, Horn K, Gilman BLC, Belden LK, Mitchell JC, Grayson KL (2019) Surveys for population persistence and *Bd* at the Northeastern range edge of the Eastern lesser siren. *Northeastern Naturalist* 26:410-419

Thompson PD, Fridell RA, Wheeler KW, Bailey CL (2004) Distribution of *Bufo boreas* in Utah. *Herpetological Review* 35:255-257

Thorpe CJ, Lewis TR, Fisher MC, Wierzbicki CJ, Kulkarni S, Pryce D, Davies L, Watve A, Knight ME (2018) Climate structuring of *Batrachochytrium dendrobatidis* infection in the threatened amphibians of the Northern Western Ghats India. *Royal Society Open Science* 5 180211

Tinsley RC, Coxhead PG, Stott LC, Tinsley MC, Piccinni MZ, Guille MJ (2015) Chytrid fungus infections in laboratory and introduced *Xenopus laevis* Populations: Assessing the risks for UK native amphibians. *Biological Conservation* 184:380-388

Tobler U, Borgula A, Schmidt BR (2012) Populations of a susceptible amphibian species can grow despite the presence of a pathogenic chytrid fungus. *PLoS ONE* 7 e34667

Todd H Fritzler JM, Kazmaier RT, Johnson JB (2019) Prevalence of *Batrachochytrium dendrobatidis* and *Ranavirus* in Western Texas, USA. *Herpetological Review* 50:505–507

Tupper TA, Streicher JW, Greenspan SE, Timm BC, Cook RP (2011) Detection of *Batrachochytrium dendrobatidis* in anurans of Cape Cod National Seashore Barnstable County, Massachusetts, USA. *Herpetological Review* 42:62-65

Tupper TA, Bozarth CA, Jones KS, Cook RP (2014) Detection of *Batrachochytrium dendrobatidis* in the Eastern Spadefoot *Scaphiopus holbrookii* at Cape Cod National Seashore Barnstable County, Massachusetts, USA. *Herpetological Review* 45:445-447

Urbina JC, Galeano SP (2011) *Batrachochytrium dendrobatidis* Detected in amphibians of the Central Andean Cordillera of Colombia. *Herpetological Review* 42:558-560

Valencia-Aguilar A, Ruano-Fajardo G, Lambertini C, Leite D. Da S, Toledo LF, Mott T (2015) Chytrid fungus acts as a generalist pathogen infecting species-rich amphibian families in Brazilian Rainforests. *Diseases of Aquatic Organisms* 114:61-67

Valencia-Aguilar A, Vital MVC, Vital MVC, Mott T (2016) Seasonality, environmental factors, and host behavior linked to disease risk in stream-dwelling tadpoles. *Herpetologica* 72:98-106

Valenzuela-Sánchez A, O’Hanlon SJ, Alvarado-Rybak M, Uribe-Rivera DE, Cunningham AA, Fisher MC, Soto-Azat C (2017) Genomic epidemiology of the emerging pathogen *Batrachochytrium dendrobatidis* from native and invasive amphibian species in Chile. *Transboundary and Emerging Diseases* 65:309-314

Van Der Hoek Y, Emmanuel F, Twahirwa JC, Tuyisenge MF, Tuyisingize D (2019) Amphibian chytrid fungus *Batrachochytrium dendrobatidis* detected at high elevations in Volcanoes National Park, Rwanda. *Herpetological Review* 50:727–729

Van Rooij P, Martel A, Nerz J, Voitel S, Van Immerseel F, Haesebrouck F, Pasmans F (2011) Detection of *Batrachochytrium dendrobatidis* in Mexican Bolitoglossine Salamanders using an optimal sampling protocol. *EcoHealth* 8:237-243

Van Sluys M, Hero J-M (2009) How does chytrid infection vary among habitats? The case of *Litoria wilcoxii* (Anura Hylidae) in SE Queensland, Australia. *EcoHealth* 6:576-583

Victoria Flechas S, Sarmiento C, Amezquita A (2012) *Bd* on the beach: High prevalence of *Batrachochytrium dendrobatidis* in the lowland forests of Gorgona Island (Colombia South America). *EcoHealth* 9:298-302

Vieira CA, Almeida CHLN, Lambertini C, Leite DD, Toledo LF. (2012) First record of *Batrachochytrium dendrobatidis* in Paraná, Brazil. *Herpetological Review* 43:93-94
Vieira CA, Toledo LF, Longcore JE, Longcore JR (2013) Body Length of *Hylodes cf. ornatus* and *Lithobates catesbeianus Tadpoles depigmentation of mouthparts and presence of Batrachochytrium dendrobatidis* are related. *Brazilian Journal of Biology* 73:195-199
Viertel B, Veith M, Schick S, Channing A, Kigoolo S, Baeza-Urrea O, Sinsch U, Lötters S (2012) The stream-dwelling larva of the Ruwenzori River Frog, *Amietia ruwenzorica* its buccal cavity and pathology of chytridiomycosis. *Zootaxa* 43-57
Villamizar-Gomez A, Forstner MRJ, Suriyamongkol T, Forks KN, Grant WE, Wang HH, Mali I (2016) Prevalence of *Batrachochytrium dendrobatidis* in two sympatric treefrog species, *Hyla cinerea* and *Hyla versicolor*. *Herpetological Review* 47:601-606
Vojar J, Havlikova B, Solsky M, Jablonski D, Ikovic V, Balaz V (2017) Distribution, prevalence, and amphibian hosts of *Batrachochytrium dendrobatidis* in the Balkans. *Slamandra* 53:44-49
Vörös J, Bosch J, Dan A, Hartel T (2013) First record of *Batrachochytrium dendrobatidis* on amphibians in Romania. North-*Western Journal of Zoology* 9:446-449
Voyles J, Vredenburg VT, Tunstall TS, Parker JM, Briggs CJ, Rosenblum EB (2012) Pathophysiology in mountain yellow-legged frogs (*Rana Muscosa*) during a chytridiomycosis outbreak. *PLoS ONE* 7 e35374
Vörös J, Price L, Donnellan SC (2011) *Batrachochytrium dendrobatidis* on the endemic frog *Litoria raniformis* in South Australia. *Herpetological Review* 42:220-223
Vörös J, Herczeg D, Fülöp A, Gál TJ, Dán Á, Harmos K, Bosch J (2018) *Batrachochytrium dendrobatidis* in Hungary: An overview of recent and historical occurrence. *Acta Herpetologica* 13:125-140
Vredenburg VT, Felt SA, Morgan EC, McNally SVG, Wilson S, Green SL (2013) Prevalence of *Batrachochytrium dendrobatidis* in Xenopus collected in Africa (1871-2000) and in California (2001-2010). *PLoS ONE* 8 e63791
Vredenburg VT, Summers AP (2001) Field identification of chytridiomycosis in *Rana muscosa* (Camp 1915) *Herpetological Review* 32:151-152
Vredenburg VT, Knapp RA, Tunstall TS, Briggs CJ (2010) Dynamics of an emerging disease drive large-scale amphibian population extinctions. *Proceedings of the National Academy of Sciences of the United States of America.* 107:9689-9694
Waddle A, Sai M, Levy J, Rezaei G, van Breukelen F, Jaeger J (2018) Systematic approach to isolating *Batrachochytrium dendrobatidis. Diseases of Aquatic Organisms* 127:243-247
Waddle AW, Levy JE, Rivera R, van Breukelen F, Nash M, Jaeger JR (2019) Population-level resistance to chytridiomycosis is life-stage dependent in an imperiled anuran. *Ecohealth* 16:701-711
Wagner N, Neubeck C, Guicking D, Finke L, Wittich M, Weising K, Geske C, Veith M (2017) No evidence for effects of infection with the amphibian chytrid fungus on populations of yellow-bellied toads. *Diseases of Aquatic Organisms* 123:55-65
Walke JB, Becker MH, Hughey MC, Swartwout MC, Jensen RV, Belden LK (2017) Dominance-function relationships in the amphibian skin microbiome. *Environmental Microbiology* 19:3387-3397
Wang H, Wang H.-N, Regassa JF, Wang X.-L (2018) Independent origin of chytrid fungus in China. *Russian Journal of Herpetology* 25:299-3010
Wang SP, Zhu W, Fan LQ, Li JQ, Li YM (2017) Amphibians testing negative for *Batrachochytrium dendrobatidis* and *Batrachochytrium salamandrivorans* on the Qinghai-Tibetan Plateau, China. *Asian Herpetological Research* 8:190-198

Warne RW, LaBumbard B, LaGrange S, Vredenburg VT, Catenazzi A (2016) Co-infection by chytrid fungus and *Ranavirus*es in wild and harvested frogs in the tropical Andes. *PLoS ONE* 11 e0145864

Watters JL, Flanagan RL, Davis DR, Farkas JK, Kerby JL, Labonte MJ, Penrod ML, Siler CD (2016) Screening natural history collections for historical presence of *Batrachochytrium dendrobatidis* in anurans from Oklahoma, USA. *Herpetological Review* 47:214-220

Watters JL, Davis DR, Yuri T, Siler CD (2018) Concurrent infection of *Batrachochytrium dendrobatidis* and *Ranavirus* among native amphibians from Northeastern Oklahoma USA. *Journal of Aquatic Animal Health* 30:291-301

Watters JL, McMillin SL, Marhanka EC, Davis DR (2019) Seasonality in *Batrachochytrium dendrobatidis* detection in amphibians in Central Oklahoma USA. *Journal of Zoo and Wildlife Medicine* 50:492-497

Weldon C, Du Preez L, Hyatt A, Muller R, Speare R (2004) Origin of the amphibian chytrid fungus. *Emerging Infectious Diseases* 10:2100-2105

Werner KJ, Heinz G, Lichtenberg J. (2006) The status of two Northern leopard frog populations in Western Montana. *Herpetological Review* 37:325-330

Whitfield SM, Geerdes E, Chacon I, Ballestero Rodriguez E, Jimenez RR, Donnelly MA, Kerby JL (2013) Infection and co-infection by the amphibian chytrid fungus and *Ranavirus* in wild Costa Rican frogs. *Diseases of Aquatic Organisms* 104:173-178

Whitfield SM, Kerby J, Gentry LR, Donnelly MA (2012) Temporal variation in infection prevalence by the amphibian chytrid fungus in three species of frogs at la Selva, Costa Rica. *Biotropica* 44:779-784

Whitfield S, Alvarado G, Abarca J, Zumbado H, Zuñiga I, Wainwright M, Kerby J (2017) Differential patterns of *Batrachochytrium dendrobatidis* infection in relict amphibian populations following severe disease-associated declines. *Diseases of Aquatic Organisms* 126:33-41

Wilson TP, Barbosa JM, Carver EA, Reynolds BR, Richards D, Salamander T, Wilson TM (2015) *Batrachochytrium dendrobatidis* prevalence in two ranid frogs on a former United States department of defense installation in Southeastern Tennessee. *Herpetological Review* 46:37-41

Wilson EA, Briggs CJ, Dudley TL (2018) Invasive African clawed frogs in California: A reservoir for or predator against the chytrid fungus? *PLOS ONE* 13 e0191537

Windstam ST, Olori JC (2014) Proportion of Hosts Carrying *Batrachochytrium dendrobatidis*, Causal Agent of Amphibian Chytridiomycosis in Oswego County, NY in (2012) Northeast. Nat. 21

Wolff BG, Conway SM, Dabney CJ, III (2012) *Batrachochytrium dendrobatidis* and *Ranavirus* in anurans inhabiting decorative koi ponds near Minneapolis Minnesota, USA. *Herpetological Review* 43:427-429

Wolff BG, Wurm E, Conway S, Kinzer K (2014) *Batrachochytrium dendrobatidis* infection rates differ over short distances between natural lakes and artificial ponds in Minnesota, USA. *Herpetological Review* 45:447-449

Woodhams DC, Bell SC, Kenyon N, Alford RA, Rollins-Smith LA (2012) Immune evasion or avoidance: Fungal skin infection linked to reduced defence peptides in Australian green-eyed treefrogs *Litoria serrata*. *Fungal Biology* 116:1203-1211

Woodhams DC, Vredenburg VT, Simon M-A, Billheimer D, Shakhtour B, Shyr Y, Briggs CJ, Rollins-Smith LA, Harris RN (2007) Symbiotic bacteria contribute to innate immune defenses of the threatened mountain yellow-legged frog *Rana muscosa*. *Biological Conservation* 138:390-398

Wunder JL, Lampazzi NM, Acre KD, Bent NJ, Canter SA, Chapman AM, Davies MA, Kashan D, Keiley JW, Macintyre RI, Milton TF, Weichler KL, Wilson MJ, Takahashi MK (2012) Promoting amphibian conservation through the college classroom: Detection of *Batrachochytrium dendrobatidis* among local amphibians. *Herpetological Conservation and Biology* 7:462-469

Yang H, Baek H, Speare R, Webb R, Park SunKyung Kim T, Lasater KC, Shin S, Son S, Park J, Min M, Kim Y, Na K, Lee H, Park SeChang (2009) First detection of the amphibian chytrid fungus, *Batrachochytrium dendrobatidis*, in free-ranging populations of amphibians on mainland Asia: Survey in South Korea. *Diseases of Aquatic Organisms* 86:9-13

Yap TA, Gillespie L, Ellison S, Flechas SV, Koo MS, Martinez AE, Vredenburg VT (2016) Invasion of the fungal pathogen *Batrachochytrium dendrobatidis* on California islands. *EcoHealth* 13:145-150

Zampiglia M, Canestrelli D, Chiocchio A, Nascetti G (2013) Geographic distribution of the chytrid pathogen *Batrachochytrium dendrobatidis* among mountain amphibians along the Italian peninsula. *Diseases of Aquatic Organisms* 107:61-68

Zellmer AJ, Richards CL, Martens LM (2008) Low prevalence of *Batrachochytrium dendrobatidis* across *Rana sylvatica* Populations in Southeastern Michigan, USA. *Herpetological Review* 39:196-199

Zhu W, Bai C, Wang S, Soto-Azat C, Li X, Liu X, Li Y (2014) Retrospective survey of museum specimens reveals historically widespread presence of *Batrachochytrium dendrobatidis* in China. *EcoHealth* 11:241-250

Zumbado-Ulate H, Bolanos F, Gutierrez-Espeleta G, Puschendorf R (2014) Extremely low prevalence of *Batrachochytrium dendrobatidis* in frog populations from neotropical dry forest of Costa Rica supports the existence of a climatic refuge from disease. *EcoHealth* 11:593-602

Zumbado-Ulate H, Nelson KN, García-Rodríguez A, Chaves G, Arias E, Bolaños F, Whitfield SM, Searle CL (2019) Endemic infection of *Batrachochytrium dendrobatidis* in Costa Rica: Implications for amphibian conservation at regional and species level. *Diversity* 11 129

Zumbado-Ulate H, García-Rodríguez A, Vredenburg VT, Searle C (2019) Infection with *Batrachochytrium dendrobatidis* is common in tropical lowland habitats: Implications for amphibian conservation. *Ecology and Evolution* 9:4917-4930
